# Clonal competition within complex evolutionary hierarchies shapes AML over time

**DOI:** 10.1038/s41467-019-14106-0

**Published:** 2020-02-05

**Authors:** Carl Sandén, Henrik Lilljebjörn, Christina Orsmark Pietras, Rasmus Henningsson, Karim H. Saba, Niklas Landberg, Hanna Thorsson, Sofia von Palffy, Pablo Peña-Martinez, Carl Högberg, Marianne Rissler, David Gisselsson, Vladimir Lazarevic, Gunnar Juliusson, Helena Ågerstam, Thoas Fioretos

**Affiliations:** 1 Division of Clinical Genetics, Department of Laboratory Medicine, Lund University, Lund, Sweden; 2 Department of Hematology, Oncology and Radiation Physics, Skåne University Hospital, Lund, Sweden

**Keywords:** Cancer stem cells, Acute myeloid leukaemia, Tumour heterogeneity

## Abstract

Clonal heterogeneity and evolution has major implications for disease progression and relapse in acute myeloid leukemia (AML). To model clonal dynamics in vivo, we serially transplanted 23 AML cases to immunodeficient mice and followed clonal composition for up to 15 months by whole-exome sequencing of 84 xenografts across two generations. We demonstrate vast changes in clonality that both progress and reverse over time, and define five patterns of clonal dynamics: Monoclonal, Stable, Loss, Expansion and Burst. We also show that subclonal expansion in vivo correlates with a more adverse prognosis. Furthermore, clonal expansion enabled detection of very rare clones with AML driver mutations that were undetectable by sequencing at diagnosis, demonstrating that the vast majority of AML cases harbor multiple clones already at diagnosis. Finally, the rise and fall of related clones enabled deconstruction of the complex evolutionary hierarchies of the clones that compete to shape AML over time.

## Abstract (web summary)

Clonal evolution and heterogeneity has strong implications for treatment response in acute myeloid leukemia. Here, the authors use patient derived in vivo modelling to highlight the complex clonal and evolutionary dynamics underpinning acute myeloid leukemia progression.

## Introduction

AML arises through successive acquisition of genetic alterations, which creates complex hierarchies of clones with different combinations of mutations and chromosomal aberrations^1,2^. However, the extent of clonal heterogeneity at diagnosis and whether relapse clones are predominantly present at diagnosis or evolve during chemotherapy remain open questions^3–14^. Patient-derived xenografts (PDXs) are the only model system that enables expansion of AML patient cells with preserved leukemia stem cell capacity^15–18^. Thus, PDXs have emerged as a critical model to study the response to chemotherapy and novel targeted therapies as well as potential resistance mechanisms, although the severe immunodeficiencies that allow human engraftment also make studies that rely on interactions between the leukemia and the immune system more challenging^19,20^. We hypothesized that serial transplantations over longer periods of time would expose inherent differences in proliferation and self-renewal capacity between competing clones and thus allow modeling of clonal dynamics in vivo. We also hypothesized that the emergence and disappearance of related clones would enable delineation of clonal hierarchies and detection of previously undetectable clones, thus unmasking clonal heterogeneity in AML.

## Results and discussion

### Patient-derived AML xenografts enable long-term modeling of clonal dynamics

**Fig. 1:**
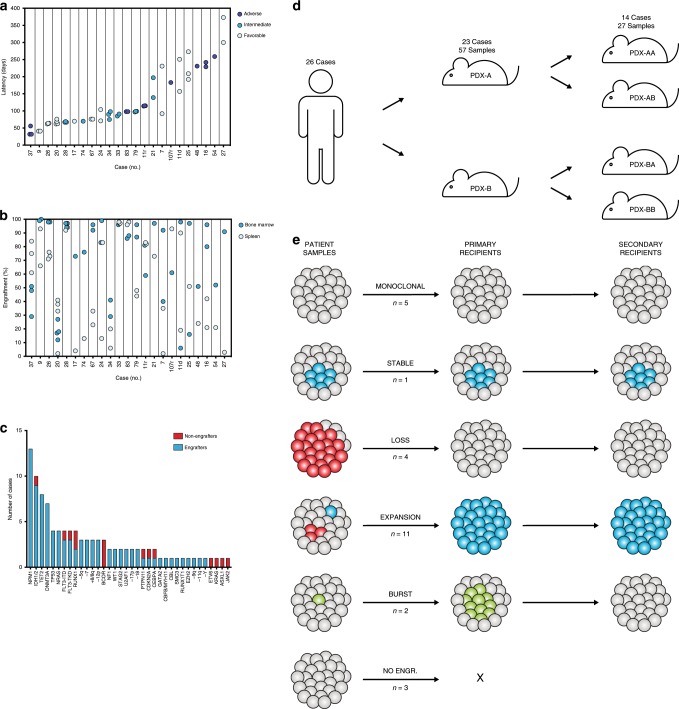
Patient-derived AML xenografts undergo extensive clonal competition across multiple generations. Latency (**a**) and engraftment levels (**b**) in primary recipient mice, identically ordered by average disease latency. Risk categories were based on the ELN 2017 guidelines. **c** Engraftment of primary AML samples based on the presence of recurrent AML mutations. **d** Schematic overview of the generation and nomenclature of PDXs. **e** Patterns of clonal dynamics identified as leukemias were transplanted to primary and secondary recipient mice. *n* denotes the number of cases that follow each pattern.

We transplanted 26 AML cases to the NSG-S mouse model^18^ and allowed engraftment to proceed until the first signs of disease. This long-term approach generated high engraftment levels from the vast majority of samples (23/26; 88%), encompassing a wide spectrum of recurrent AML aberrations (Fig. 1a–d, Table 1). All 26 patient samples and 84 xenografts were subjected to whole-exome sequencing (WES) to track clonal dynamics based on somatic mutations, copy number alterations (CNA) and copy-neutral losses of heterozygosity, making this the largest cohort of paired AML and xenograft samples to date^9,21–23^. In contrast to previous work, we also allowed clonal evolution to proceed until the development of disease in both primary and secondary recipients, yielding combined latencies of up to 15 months.

**Table 1:** Patient characteristics of transplanted AML cases.

Case	Tissue	Age	Gender	WBC count	FAB type	ELN 2017 risk	Recurrent mutations	Karyotype
AML-4	BM	39	Female	3,4	M4	Adverse	FLT3-ITD	NK
AML-7	BM	60	Female	8,2	M4	Favorable	FLT3-TKD, IDH2, NPM1, WT1	NK
AML-9	PB	48	Female	25,3	M5	Favorable	DNMT3A, TET2, NPM1	NK
AML-10	PB	72	Male	21,1	M4	Adverse	ASXL1, IDH2, JAK2, RUNX1	inv(9)(p11q12)
AML-11d	PB	64	Female	58,0	M5	Favorable	CDKN2A, DNMT3A, KRAS, NPM1, TET2, U2AF	+8
AML-11r	BM	66	Female	15,6	M2	Intermediate	DNMT3A, RUNX1, TET2, U2AF	+8
AML-16	BM	64	Male	24,9	M1	Favorable	FLT3-TKD, NPM1, STAG2, TET2	NK
AML-17	PB	73	Male	12,1	M5	Intermediate	DNMT3A, GATA2, NPM1, NRAS	NK
AML-20	PB	41	Male	39,1	M4	Favorable	CBFB/MYH11, NRAS	inv(16)(p13q22)
AML-21	BM	70	Male	76,0	M2	Intermediate	BCOR, IDH2, NRAS, STAG2	NK
AML-24	BM	72	Male	5,5	M2	Favorable	IDH2, NPM1	NK
AML-25	BM	86	Male	2,2	M2	Favorable	NPM1, TET2	−Y
AML-26	PB	59	Female	91,9	M4	Favorable	DNMT3A, IDH2, NPM1	NA
AML-27	PB	59	Male	47,0	M1	Intermediate	IDH1, NPM1	NK
AML-28	BM	67	Male	37,5	M2	Intermediate	FLT3-ITD, NPM1, TET2	NK
AML-33r	PB	43	Female	83,3	M4	Intermediate	FLT3-ITD, IDH1, NPM1	NK
AML-34	BM	50	Female	35,0	M5	Intermediate	CEBPA, DNMT3A, IDH1, NRAS	NK
AML-37	BM	73	Male	19,0	M5	Adverse	CDKN2A, FLT3-TKD, TP53	Complex
AML-47r	BM	35	Male	24,0	M1	Adverse	BCOR, FLT3-TKD, PTPN11, RUNX1	NK
AML-48	PB	82	Female	58	M2	Adverse	TP53	Complex
AML-54	BM	58	Male	2,2	M2	Adverse	RUNX1, EZH2, BCOR, ETV6, CEBPA	−7, +8
AML-67	PB	63	Female	15,1	M4	Favorable	DNMT3A, NPM1, RUNX1T1, TET2	NK
AML-74	PB	47	Male	4,9	M2	Intermediate	IDH1	del(12)(p11p13)
AML-79	PB	58	Male	73,0	M4	Intermediate	FLT3-ITD, IDH2, NPM1	NK
AML-83	BM	72	Male	25,9	M2	Adverse	NF1, TET2, TP53	Complex
AML-107r	BM	42	Female	2,5	M2	Adverse	TP53, CBL	Complex

Cancer cell clones are defined as cell populations containing unique sets of genetic aberrations. Thus, we first identified clones based on all detected mutations using the PyClone algorithm^24^. With this approach, we found that the vast majority of cases (21/23; 91%) underwent changes in clonal composition from the patient samples to the derived xenografts, with 19 of 23 cases (83%) exhibiting subclonal expansion in vivo and 13 of 23 cases (57%) exhibiting subclonal reduction or loss (Supplementary Data 1−22). However, most of these clones were marked by presumed passenger or even synonymous mutations with limited or no functional significance. Hence, we thenceforth restricted the analysis to clones defined by recurrent AML mutations^25^ or CNA. For the subsequent analyses, cells with additional nonrecurrent presumed passenger mutations were thus not considered to constitute distinct subclones. With regard to clones with AML-associated aberrations, we identified five patterns of clonal dynamics in AML PDXs herein referred to as: Monoclonal, Stable, Loss, Expansion and Burst (Fig. 1e).

### AML xenografts undergo extensive clonal competition

**Fig. 2:**
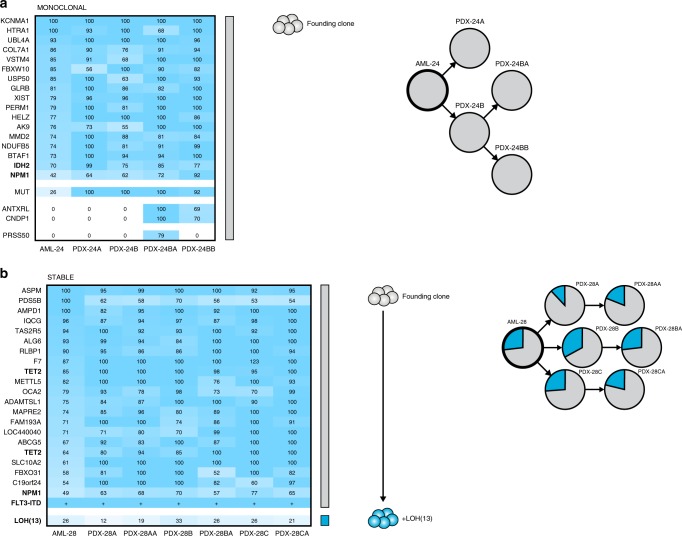
The clonal composition is maintained in a minority of AML xenografts. **a** A representative AML case with the Monoclonal pattern of clonal dynamics, where the patient sample only contains one detectable clone with AML-associated mutations or CNAs and this clone constitutes the entire xenografts. In AML-24, a single clone with *IDH2* and *NPM1* mutations gave rise to both the two primary and the two secondary xenografts, differing only in terms of nonrecurrent presumed passenger mutations. Left, the percentage of cells in patient samples and corresponding xenografts estimated to carry each genetic aberration, based on variant allele frequencies of identified mutations and b-allele frequencies of copy number alterations and copy-neutral losses of heterozygosity. Colored bars indicate defining mutations for each clone. Clones are represented by the same color throughout each panel. Middle, inferred clonal hierarchy. Right, proportions of each clone at diagnosis (AML, thick circles) and in xenografts (PDX, thin circles). Clones were defined by the presence of one or more recurrent AML mutations, CNAs or losses of heterozygosity (indicated in bold). **b** The only AML case with the Stable pattern of clonal dynamics, where clones in the patient sample retain their relative proportions in the xenografts. In AML-28, a subclone with loss of heterozygosity of chromosome 13 maintained its frequency from the patient sample in all three primary and three secondary xenografts. The presence of *FLT3*-ITD is denoted by +, as the rearrangement in this case is detectable but not quantifiable by WES.

The composition of clones with different AML-associated alterations was found to change significantly from the patient sample to the xenografts in as many as 17 of 23 cases (74%), thereby enabling inference of clonal hierarchies and detection of rare clones. The remaining 6 of 23 AML cases (26%) exhibited the same clonal composition at diagnosis and in the xenografts. In five of these cases, the patient sample and the PDXs contained the same single AML clone, referred to as Monoclonal, only differing in terms of presumed passenger mutations (Fig. 2a; additional cases in Supplementary Fig. 1). In one case, a minor subclone was maintained at a low frequency in vivo, referred to as Stable (Fig. 2b).

**Fig. 3:**
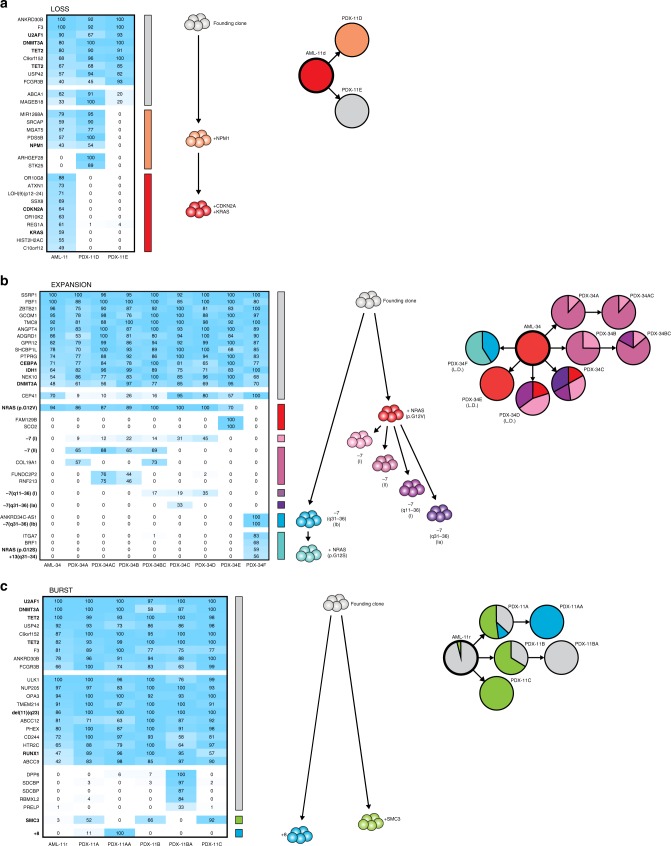
The clonal composition changes in the majority of AML xenografts. **a** A representative AML case with the Loss pattern of clonal dynamics, where a subclone in the patient sample is reduced or completely lost in the xenografts. In AML-11d, the dominant clone in the patient sample, with *CDKN2A* and *KRAS* mutations, was lost in both xenografts, resulting in engraftment with one of two parental clones. Left, the percentage of cells in patient samples and corresponding xenografts estimated to carry each genetic aberration, based on variant allele frequencies of identified mutations and b-allele frequencies of copy number alterations and copy-neutral losses of heterozygosity. Colored bars indicate defining mutations for each clone. Clones are represented by the same color throughout each panel. Middle, inferred clonal hierarchy. Right, proportions of each clone at diagnosis and in PDXs. Clones were defined by the presence of one or more recurrent AML mutations, CNAs or losses of heterozygosity (indicated in bold). L.D. denotes samples transplanted at minimal cell dose based on limiting dilution analysis. **b** A representative AML case with the Expansion pattern of clonal dynamics, where a subclone in the patient sample expands to constitute the entirety of the xenografts. In AML-34, clones with one of two different NRAS mutations and five different partial or complete losses of chromosome 7, which were all undetectable at diagnosis, expanded to generate the xenografts. The heterozygous *CEP41* mutation is present in the founding clone but located on chromosome 7 and thus lost in the −7 (II) clone but retained in the −7 (I) clone. I and II denote the two alleles of chromosome 7, whereas a and b represent distinct genetic events. **c** A representative AML case with the Burst pattern of clonal dynamics, where a subclone in the patient sample expands in primary xenografts but is lost in secondary xenografts. In AML-11r, a small subclone with an *SMC3* mutation transiently expanded to make up the majority of both primary recipients but was lost in the two secondary recipients. The +*8* aberration was detected at very low frequency in the diagnostic sample by routine clinical karyotyping.

**Fig. 4:**
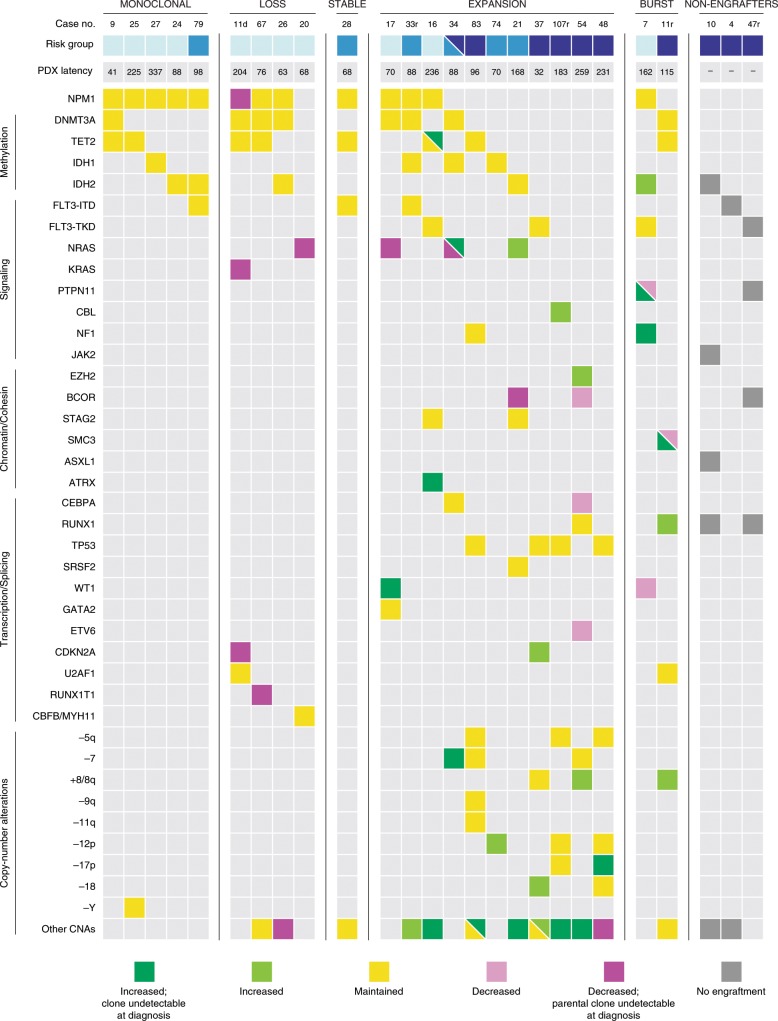
Clonal competition in AML xenografts unmasks rare clones and correlates with genetic risk. Recurrent genetic alternations of all AML cases and changes in allele frequencies from patient samples to xenografts. Dark green: mutation with increased frequency that was undetectable at diagnosis. Light green: mutation with increased frequency. Yellow: mutation with maintained frequency. Light purple: mutation with reduced frequency. Dark purple: mutation with reduced frequency, revealing a parental clone that was undetectable at diagnosis. Gray: mutation in nonengrafting sample. CNA copy number alteration. Relapse samples are denoted by “r” after the case number. Risk category classification based on the ELN 2017 guidelines. Light blue: favorable. Medium blue: intermediate. Dark blue: adverse. The dual coloring for AML-34 reflects the classification as intermediate based on information from the diagnostic sample alone and the classification as adverse when incorporating information derived from xenografts.

In 4 of 23 AML cases (17%), the major clone at diagnosis was lost or reduced in the xenografts, giving way to a parental clone containing all but one or two of the AML mutations, referred to as Loss (Fig. 3a; Supplementary Fig. 2). The presence of these parental clones at diagnosis could not be predicted based on sequencing of the patient samples alone, as the mutations defining the lost subclones had allele frequencies as high as those pertaining to the parental clones. Thus, the Loss pattern unveiled clonal diversity at diagnosis and identified late events during leukemogenesis, including mutations in *CDKN2A* and *RUNX1T1*. Strikingly, four cases lost clones with *NRAS* or *KRAS* mutations (Fig. 4), mirroring a common clinical scenario where *RAS* mutations are lost from diagnosis to relapse^7^. In multiple cases, the subclones did not start to decrease until the second generation of xenografts, suggesting that certain late mutations may allow or even promote initial expansion in vivo but eventually exhaust the leukemia stem cell population.

In 11 of 23 AML cases (48%), the xenografts emanated from a minor subclone in the diagnostic sample, referred to as Expansion (Fig. 3b; Supplementary Fig. 3). The expanding clones had average allele frequencies of only 5% in the patient samples, and in three cases the clones were undetectable at diagnosis and only revealed by expansion in vivo (AML-34: Fig. 3b, AML-16: Supplementary Fig. 3a and AML-17: Supplementary Fig. 3b). In AML-34, clonal expansion revealed the presence of four separate subclones with losses of different segments and alleles of chromosome 7, none of which were detected by WES (81×) or routine clinical karyotyping at diagnosis. Interestingly, one xenograft carried neither the NRAS (p.G12V) mutation nor any of these losses of chromosome 7 but instead revealed the presence of another clone carrying a different NRAS mutation (p.G12S) and a different partial loss of chromosome 7. These strikingly parallel evolutionary trajectories suggest that the emergence of specific mutations in cancer may be driven less by stochastics and more by the intrinsic and extrinsic evolutionary pressures on the individual leukemia. It also indicates that certain leukemias may harbor pools of distinct but genetically similar clones. Loss of chromosome 7 is a risk-defining alteration according to the ELN 2017 guidelines^26^, and provided that this holds true also at low frequencies, detection of these subclones at diagnosis would have changed the risk assessment from intermediate to adverse. This suggests that AML patients may harbor unexpected clonal complexity with clinical implications, including rare clones with CNAs that are not detectable by sequencing at diagnosis. The expansion of minor subclones led to complete loss of parental clones and sibling clones carrying other recurrent AML mutations, thus unmasking clonal hierarchies. The losses of the *BCOR* mutations in AML-21 and AML-54 (Supplementary Fig. 3c and Fig. S3i), the *WT1* mutation in AML-7 (Supplementary Fig. 4), the *CEBPA* and *ETV6* mutations in AML-54 (Supplementary Fig. 3i) as well as the *NRAS* mutations in AML-17 and AML-34 (Supplementary Fig. 3b and Fig. 3b) demonstrate not only their late acquisition but also evolutionary branching with multiple clones containing mutually exclusive driver mutations, something that cannot be inferred from sequencing of diagnostic samples alone.

In 2 of 23 AML cases (9%), rare clones with allele frequencies as low as the background at diagnosis expanded in primary xenografts but were lost in secondary recipients, referred to as Burst (Fig. 3c; Supplementary Fig. 4). This is the first report to demonstrate that changes in clonal composition can be reversed after secondary transplantation, suggesting that AML cells undergo continuous clonal competition across multiple generations in vivo and that certain genetic aberrations may confer initial expansion at the expense of long-term self-renewal.

### AML PDXs are genetically stable with moderate reproducibility

In many cases, the emergence of even very rare clones was notably consistent between replicate xenografts, as with the *NRAS* clone in AML-21, the −7 clones in AML-34 and the transient burst of the *SMC3* clone in primary xenografts from AML-11r. However, certain patterns were less consistent, such as the loss of the *NRAS* clone observed in two of five xenografts from AML-20, and the varying expansion of the *NF1*, *FLT3* and *PTPN11* clones in different xenografts from AML-7.

To further dissect the reproducibility, we performed limiting dilution analysis of two patient samples with substantial changes in clonal composition. We then analyzed the xenografts generated by the minimal required cell doses, which should be the most susceptible to random variation. In AML-21, all five xenografts displayed the same expansion of the minor *NRAS* clone seen in the other PDXs (Supplementary Fig. 3a). However, the three PDXs generated from AML-34 all displayed different clonal compositions (Fig. 3b).

Thus, engraftment seems to be largely dictated by clonal fitness but still subject to stochastic variation, especially at lower cell doses. However, this variability should decrease over time as clonal heterogeneity is progressively reduced and more clones are permanently lost. We would thus speculate that the most consistent clonal contribution is obtained with high cell numbers from serially passaged xenografts.

Despite the striking changes in clonal composition, AML PDXs were found to be remarkably genetically stable, with fewer acquired mutations than the commonly used retroviral *MLL-AF9* mouse model^27,28^ and fewer novel CNAs than PDXs from other tissues^29^. At least 15 of the 19 recurrent AML alterations that emerged in the xenografts could be retraced to the diagnostic samples, and WES only identified 0.44 passenger mutations per passage, with a majority of the xenografts not gaining any novel mutations despite an average latency of 110 days in vivo.

### AML xenografts can reveal but not predict relapse clones

To determine if clonal evolution in vivo mirrors the development from diagnosis to relapse, we analyzed matched samples from seven AML cases (Supplementary Fig. 5). In two cases, the clonal dynamics in patients and xenografts converged. In AML-7, the xenografts revealed the presence of a subclone with an *NF1* mutation that was otherwise undetectable at diagnosis and emerged as the major clone at relapse. In AML-11, the engrafting clones mirrored the loss of the *CDKN2A*, *KRAS* and *NPM1* mutations seen at relapse but did not recapitulate the gain of a *RUNX1* mutation and del(11)(q23) observed at relapse. However, in four cases the clonal dynamics diverged and the clones that emerged or persisted in vivo were lost at relapse. One case displayed no change in clonal composition. Thus, we show that AML PDXs can reveal rare relapse clones in diagnostic samples but in accordance with previous work^21^, we find that the clonal evolution in xenografts does not consistently mimic that from diagnosis to relapse. This may be due to differences in the human and mouse microenvironments or the lack of selective pressure from chemotherapy in the PDX model. In terms of engraftment characteristics, we did not see a difference between the 20 diagnostic samples and the three relapse samples transplanted in our cohort but observed that all three relapse samples contained multiple clones and exhibited expansion of minor subclones in vivo, possibly contributing to the increased aggressiveness at relapse.

### Clonal dynamics in AML PDXs correlates with genetic risk

We also found a correlation between clonal dynamics and genetic risk. All six cases (6/6; 100%) with adverse risk genetics were found to contain subclones at diagnosis that expanded in vivo, compared to four of the six cases (4/6; 67%) with intermediate risk and only three of the 11 cases (3/11; 27%) classified as favorable risk (*p* = 0.008; Fisher’s exact test, two-sided) (Fig. 4). Thus, clonal and functional heterogeneity could contribute to the poor prognosis^30,31^ as well as the traditionally high engraftment capacity^22,32^ of adverse and intermediate risk AML patients. Conceivably, leukemias with large repertoires of potentially expansive clones would more likely contain or be able to generate clones with the capacity to withstand chemotherapy and re-emerge to cause relapse. The observed difference in genetic risk largely stems from differences in *NPM1* mutation status, with nine of ten *NPM1*^*wt*^ cases (90%) but only 4 of 13 *NPM1*^*mut*^ cases (31%) displaying expansion of minor subclones in vivo. It is thus also possible that *NPM1*-mutated AML is less prone to clonal branching and/or expansion, a feature that could potentially contribute to the relatively favorable prognosis of this subtype. In contrast to genetic risk, we did not see a correlation between clonal dynamics and latency, indicating that the expansion rates of the respective clones are more impactful than their frequencies.

### Clonal dynamics is validated by single-cell sequencing

Single-cell sequencing has emerged as a powerful tool to decipher cancer cell heterogeneity. Transcriptional analysis has identified and characterized different types of functionally distinct AML cells^33,34^, and single-cell genotyping has unmasked clonal complexity^35–37^ with implications for both disease progression^38^ and treatment resistance^39,40^. However, single-cell DNA sequencing is dependent on amplification of small amounts of DNA, which generates both false-positives and false-negatives^41^, making it difficult to identify rare clones at diagnosis that are readily detectable upon expansion in xenografts.

We performed scRNA-seq with the 10× Genomics platform on one such case and identified monosomy 7 in the diagnostic sample and two xenografts from AML-34, where clones with losses of either allele emerged in different frequencies. This analysis confirmed the expansion of clones with monosomy 7 in the xenografts, with PDX-34B dominated by the −7(II) clone and PDX-34C only containing the −7(I) clone and clones with partial losses of chromosome 7 that were too small to detect by this method (Supplementary Fig. 6). As with WES, the clones with monosomy 7 were not reliably detectable by scRNA-seq at diagnosis, as the potentially positive cells were rare and of low confidence (frequency: 1.1%; expected background rate: 0.2%), and did not form a distinct cluster in the gene expression space. This analysis thus confirmed the clonal dynamics observed by WES and underscored the potential of PDXs to uncover otherwise undetectable clonal heterogeneity in cancer.

### AML xenografts unmask clonal heterogeneity at diagnosis

By generating and sequencing xenografts, we could infer the presence of multiple clones with distinct sets of recurrent AML mutations or CNAs in 18 of 23 AML cases (78%) at diagnosis. This is higher than what was recently seen in a smaller cohort (4/11; 36%)^22^ of single primary recipient mice by a 19-gene panel, and higher than what has been reported based on whole-genome sequencing (WGS) of diagnostic samples without xenotransplantation (27/50; 54%)^42^ and WES of matched diagnosis and relapse patient samples (23/50; 46%)^7^. Strikingly, at least 11 of 23 cases (48%) contained clones that were present at diagnosis but only revealed by xenografting, thereby also identifying five recurrent AML genetic alterations (*NF1*, *PTPN11*, *SMC3*, *TET2* LoH and −7) that were not detected in the clinical diagnostic samples (Fig. 4). The presence of these clones at diagnosis could be inferred from detection in multiple independent lines of xenografts (e.g. AML-34; Fig. 3b), an approach that cannot be employed in studies comparing diagnostic and relapse samples. By applying this principle, we also identified rare diagnostic clones with CNAs, which are inherently difficult to detect by retrospective deep sequencing. Previous work has demonstrated the expansion of minor subclones in cases known to have multiple detectable clones^21,23^. However, the frequent emergence of undetectable subclones in seemingly monoclonal patients has not previously been shown and suggests that AML harbors more clonal heterogeneity at diagnosis than is detectable by standard methods. In the 11 cases with unmasked clones, the changes in clonal composition also revealed information about clonal hierarchies not discernable at diagnosis, including genetic aberrations shown to be acquired late during leukemogenesis, branching events and mutations occurring in separate subclones (e.g. AML-21: Supplementary Fig. 3b and AML-7: Supplementary Fig. 4). Thus, our sequencing of PDXs shows AML patients to harbor more clonal heterogeneity than previously described. The presence of rare previously undetectable clones at diagnosis also raises the possibility that genetic changes from diagnosis to relapse to a larger extent than previously believed may result from competition between rare pre-existing diagnostic clones rather than clonal evolution throughout chemotherapy and remission.

In this study, we demonstrate that AML generally harbors multiple clones already at diagnosis, including rare clones that are not detectable at diagnosis but only revealed upon serial transplantation. We show that individual clones are genetically stable but undergo extensive and reversible clonal competition over time. We thus conclude that AML harbors a high degree of clonal and functional heterogeneity, with implications for disease biology and future therapeutic strategies in AML.

## Methods

### Patient-derived xenografts

Aspects of the study involving patient cells were approved by the Swedish Ethical Review Authority. Informed consent was obtained and all work was conducted in compliance with all relevant ethical regulations for work with human participants. Aspects of the study involving research animals were approved by the regional Animal Ethics Committee of Malmö/Lund and complied with all relevant ethical regulations for animal testing and research. Bone marrow and peripheral blood samples from AML patients were collected at the Department of Clinical Genetics, Skåne University Hospital after written informed consent. Mononuclear cells were prepared by lymphoprep separation (GE Healthcare) and viably frozen. For primary and secondary transplantations, cells were thawed and T cells depleted by either CD3 microbead separation (Miltenyi Biotec) or treatment with the OKT3 anti-CD3 antibody (BioXCell). Except where otherwise noted, a total of ≥5 million cells were transplanted by tail vein injection to sublethally irradiated NOD.Cg-Prkdc^scid^Il2rg^tm1Wjl^/SzJ-SGM3 (NSG-S) mice (250 cGy), a substrain of the NSG mouse overexpressing hGM-CSF, hIL-3 and hSCF (Jackson Laboratory). Mice were euthanized upon signs of serious illness or 1 year after transplantation, whereupon leukemic engraftment, defined as the percentage of hCD45^+^CD33^+^CD3^−^CD19^−^ cells, was assessed by flow cytometry on an LSR Fortessa (BD Biosciences). For limiting dilution experiments, 10^3^, 10^4^, 10^5^ and 10^6^ cells were similarly transplanted and sacrificed after 16 weeks. For sequencing, leukemia cells from PDX, defined as hCD45^+^CD33^+^CD3^−^CD19^−^, were sorted by flow cytometry on a FACS Aria Fusion (BD Biosciences). The gating strategy is detailed in Supplementary Fig. 7.

### Whole-exome sequencing

Somatic mutations, CNAs and losses of heterozygosity were detected by WES. DNA was isolated from patient bone marrow or peripheral blood and from sorted CD45^+^CD33^+^ xenograft cells by the DNeasy Blood & Tissue Kit (Qiagen). WES libraries were produced using the Nextera rapid capture exome kit (Illumina) or the human core exome kit (Twist Bioscience) and sequenced by paired end 2 × 150 bp sequencing on a NextSeq 500 (Illumina). After WES, adapter sequences were trimmed from the reads using cutadapt 1.9.1. To minimize the impact of sequence reads of murine origin, the reads were aligned to both human reference genome hg19 and murine reference genome mm10 using bwa mem^43^. Reads of murine and ambiguous origin were then removed using disambiguate^44^. After alignment and murine read removal, the median coverage of targeted regions was 113×. Somatic mutations were detected using strelka 2.8.4^45^ and freebayes 1.1.0^46^ followed by a custom filter for identifying somatic variants. A freebayes somatic score was calculated as the sum of the log10-scaled genotype likelihoods (GL) for the normal sample to differ from the alternative genotype and the tumor sample to differ from the normal genotype (GL_NS,NGT_ − GL_NS,TGT_ + GL_TS,TGT_ − GL_TS,NGT_, where NS = normal sample, TS = tumor sample, NGT = normal genotype, and TGT = tumor genotype). Variants with a strelka somatic snv score (QSS) above 140, strelka somatic variant score (QSI) above 100, or freebayes somatic score above 90 were considered somatic variants. Variants suspected to originate from murine reads not removed using the read filter strategy were identified and removed by comparing against a mouse variant blacklist^47^ created by aligning whole genome sequencing reads from the Mouse Genome Project^48^ to human genome hg19. All positions with remaining somatic variants were re-genotyped in all samples from this patient using freebayes 1.1.0 to determine the frequency in all relevant samples. Complete lists of somatic variants and their frequencies in each sample are presented as Supplementary Data 23−44. Variant allele frequencies of recurrently mutated genes were validated in patient samples using a Nextera rapid capture custom kit targeting 109 genes recurrently mutated in AML that was sequenced to a median coverage of 587× (Supplementary Data 23−44). Copy number aberrations and loss of heterozygozity variants were detected from whole-exome data using cnvkit 0.9.1^49^. Germline variants fulfilling the following criteria were used to calculate SNV b-allele ratio for loss of heterozygosity detection: (1) freebayes quality (QUAL) > 300); (2) combined coverage within samples from one patient >200 and (3) only two detectable genotypes with allele frequency between 0.4 and 0.6 in the germline sample. Complete lists of CNAs in each sample are presented as Supplementary Data 45−58.

### Single-cell sequencing

For single-cell sequencing, CD45^+^CD33^+^ cells from patient and xenograft samples were sorted as described above. Single-cell 3′ RNA-seq libraries were prepared using the Chromium Single-cell 3′ reagent kit v3 (10× genomics). Single cells were separated into gel beads-in-emulsion (GEMs) using the Chromium controller (10× genomics) and full-length transcripts containing cellular and unique molecular barcodes were produced inside individual GEMs, after which the GEMs were broken and the barcoded transcripts amplified using PCR. The amplified full-length cDNA was fragmented enzymatically, the fragment ends were repaired and A-tailed, and Illumina-compatible adaptor oligos were ligated onto the fragments. The fragments where then amplified and extended to include full-length sequencing adaptors by PCR. The final libraries were sequenced on a Nextseq 500 (Illumina) with 34,000−36,000 reads/cell. Single-cell gene expression data were produced from the sequencing data using the cell ranger pipeline (10× genomics). Genotyping and detection of loss of chromosome 7 in the single cells was performed by genotyping 54 single nucleotide polymorphisms (SNPs) with coverage in the single-cell data and known assignment to either the maternal or paternal chromosome 7 as determined by exome sequencing in samples with loss of chromosome 7. The SNPs were genotyped in each cell using vartrix^50^, the genotype data were pooled across SNPs and a single read containing a genotype assigned to either chromosome 7 was interpreted as presence of that chromosome 7 in that cell. A minimum of six reads from a single chromosome 7 without any reads from the other chromosome 7 was interpreted as loss of chromosome 7. Gene expression data and chromosome 7 genotyping data were visualized using Seurat v3^51^.

### Clonal analysis

Analysis of clonal composition based on both presumed driver and passenger mutations was performed using the PyClone algorithm^24^. The analysis of clones defined by recurrent AML aberrations was performed manually and validated by PyClone. First, the percentage of cells carrying each genetic aberration was calculated by adjusting the variant allele frequency for ploidy, based on the copy number analysis. Percentages were capped at 100%. The clonal hierarchy was then determined as the solution to the clonal structure that required the least diversion from the observed allele frequencies. In cases with ambiguous clonal hierarchies, the presence of potential subclones was determined by comparing the variant allele frequencies of the potential subclone and the potential parental clone using the Mann−Whitney *U* nonparametric statistical test with a threshold of *p* < 0.05. For AML-34, the identification of multiple clones with losses of different segments of the two alleles of chromosome 7 was performed by quantification of the heterozygous somatic mutation in *CEP41* as well as SNVs on chromosome 7 that differed between the two alleles and thus displayed different frequencies in xenografts dominated by different clones.

## Supplementary information

Supplementary Data 58

Supplementary Information

Description of Additional Supplementary Files

Supplementary Data 1

Supplementary Data 2

Supplementary Data 3

Supplementary Data 4

Supplementary Data 5

Supplementary Data 6

Supplementary Data 7

Supplementary Data 8

Supplementary Data 9

Supplementary Data 10

Supplementary Data 11

Supplementary Data 12

Supplementary Data 13

Supplementary Data 14

Supplementary Data 15

Supplementary Data 16

Supplementary Data 17

Supplementary Data 18

Supplementary Data 19

Supplementary Data 20

Supplementary Data 21

Supplementary Data 22

Supplementary Data 23

Supplementary Data 24

Supplementary Data 25

Supplementary Data 26

Supplementary Data 27

Supplementary Data 28

Supplementary Data 29

Supplementary Data 30

Supplementary Data 31

Supplementary Data 32

Supplementary Data 33

Supplementary Data 34

Supplementary Data 35

Supplementary Data 36

Supplementary Data 37

Supplementary Data 38

Supplementary Data 39

Supplementary Data 40

Supplementary Data 41

Supplementary Data 42

Supplementary Data 43

Supplementary Data 44

Supplementary Data 45

Supplementary Data 46

Supplementary Data 47

Supplementary Data 48

Supplementary Data 49

Supplementary Data 50

Supplementary Data 51

Supplementary Data 52

Supplementary Data 53

Supplementary Data 54

Supplementary Data 55

Supplementary Data 56

Supplementary Data 57

## Data Availability

The datasets generated during the current study fall under the GDPR regulations for sharing of personal data and will therefore be made available in the EGA-SE depository upon its completion. Until then, the datasets are available from the corresponding authors upon request through the following DOIs: 10.17044/NBIS/G000015 (WES dataset) and 10.17044/NBIS/G000016 (scRNA-seq dataset).
