# Supplementary material for: Clonal competition within complex evolutionary hierarchies shapes AML over time
Source: Nat Commun. 2020 Feb 5;11:579. doi: 10.1038/s41467-019-14106-0 (PMC7002407; doi:10.1038/s41467-019-14106-0)
Supplement: Supplementary file 2 — Supplementary Information [file 41467_2019_14106_MOESM2_ESM.pdf]

Supplementary Information

Clonal competition within complex evolutionary hierarchies shapes AML over time

Sandén et al.

Supplementary Figures

a

MONOCLONAL

	AML-79	PDX-79A	PDX-79B	PDX-79C
ZIC2	100	100	96	100
ITIH3	97	96	100	94
GADL1	97	98	100	86
IL31RA	97	100	100	100
AGXT	96	82	100	73
CCDC54	96	100	100	100
DSCAML1	93	84	91	66
CCDC168	90	100	88	100
ENTPD4	89	78	100	96
**IDH2**	89	75	91	82
RYR2	88	88	100	100
ESPNL	87	88	85	74
IRF5	86	99	100	46
SORCS2	85	83	92	82
KRT39	84	90	84	71
NPAP1	83	91	91	74
OR4B1	80	83	94	58
TGS1	78	94	86	76
XDH	77	86	80	85
MYO5C	75	100	100	77
**NPM1**	74	83	62	85
SLC6A1	71	71	84	74
COL15A1	67	86	98	100
SIGLEC1	64	85	75	100
ADAM29	88	85	100	78
WASH2P	83	82	82	71
NHEJ1	74	91	89	69
**FLT3-ITD**	68	72	86	84

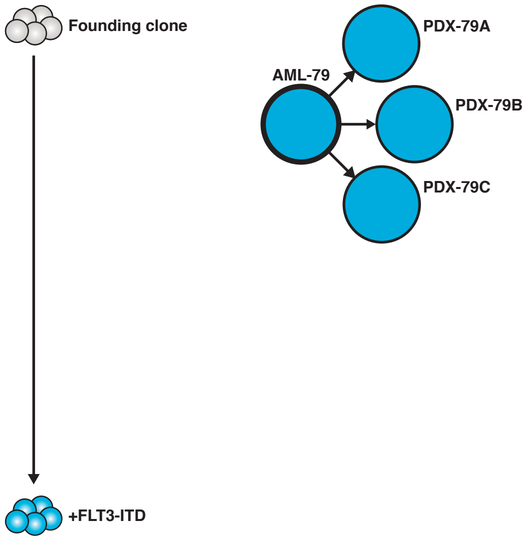

b

MONOCLONAL

	AML-25	PDX-25B
MYH3	100	100
LRP4	100	91
MCM2	99	95
TRPA1	96	100
SCARA3	94	78
GRM4	94	89
ADAM18	93	94
PSMA8	93	85
C5orf60	91	100
DOCK8	89	100
NRP2	88	100
OR4C15	88	100
RGS12	88	99
MAP2K4	85	78
CD37	84	100
SLC27A4	83	100
FRMD3	82	89
**TET2**	82	96
**-Y**	75	83
SIK3	67	95
**NPM1**	53	77
MON1A	0	71
RGPD4	0	68
ALDH4A1	0	53

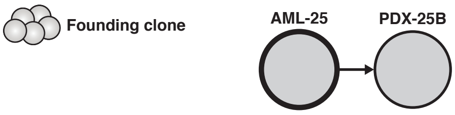

c

MONOCLONAL

	AML-27	PDX-27A
TBC1D9B	100	100
FMN1	100	100
MRPL27	99	100
NLRP12	98	100
SPTA1	97	99
UBR2	96	84
FGF2	94	100
MCM3AP	94	100
CCDC160	93	100
ISLR2	91	72
ANAPC2	89	84
YARS2	88	100
DNAH3	85	100
OR5H15	83	90
PTPRR	79	98
**IDH1**	73	72
**NPM1**	51	58
SLC5A1	22	0
MTTP	1	67
NUDCD3	0	67

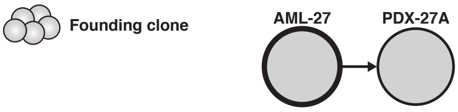

d

MONOCLONAL

	AML-9	PDX-9B	PDX-9C
PRKCG	78	71	81
FARS2	73	75	95
NUDT6	70	87	84
TMEM35	69	90	97
**DNMT3A**	69	98	100
**TET2**	69	95	100
MAPK11	67	100	100
FBN2	67	100	100
ENPP3	67	85	92
PCDHGB1	65	97	99
ECHDC1	64	93	95
OR5K2	60	100	91
ANK2	59	84	96
APC	57	100	100
CACNA1B	57	99	84
**TET2**	55	89	97
DVL1	53	100	74
CCBL2	43	71	94
GALNT6	35	100	98
**NPM1**	32	87	84
GPR55	32	94	91
ANKRD20A4	27	87	81
KMT2C	2	56	24
FAM72C	1	39	42

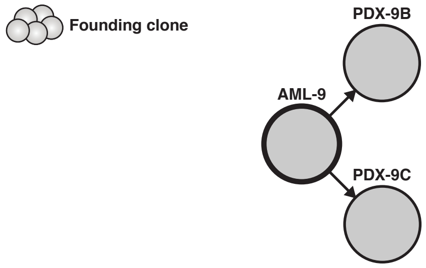

Supplementary Figure 1. Additional xenografts with the Monoclonal pattern. (a-d) Four additional cases with the Monoclonal pattern of clonal dynamics.

a

LOSS

Gene	AML-67	PDX-67A	PDX-67AA	PDX-67B	PDX-67BA
ASPHD2	100	97	100	83	88
SPEN	91	100	100	91	100
GCNT2	90	93	94	89	78
SLC7A10	89	93	90	60	78
GRAMD1C	88	88	100	99	88
OR56A1	86	100	81	98	100
MEIS1	85	87	100	100	100
C3	84	73	89	64	93
PTAFR	83	100	100	93	100
DCLK2	80	100	96	82	75
**DNMT3A**	80	97	100	97	100
DAZAP1	79	91	100	100	100
BPIFC	76	100	100	100	100
**TET2**	76	100	96	94	100
ASPG	74	99	99	100	90
SPAG1	72	90	100	100	68
FARP1	69	100	84	100	96
OR4M1	65	94	49	69	80
**NPM1**	57	61	71	66	68
FCGBP	53	95	98	93	97
PRRG2	52	67	86	56	84
PGAP1	48	88	69	90	100
NRG2	0	0	0	0	71
CTNNA2	0	0	0	0	61
ZNF182	0	0	0	0	59
ATP7B	0	0	0	0	46
AAAS	0	1	0	0	46
BASP1P1	0	0	0	0	41
F10	0	0	0	0	37
PLEKHN1	89	100	97	85	40
PCLO	87	96	91	83	49
BMP5	86	99	100	80	25
**RUNX1T1**	84	81	100	95	30
FOXP1	83	93	100	97	40
PKHD1L1	80	100	100	93	34

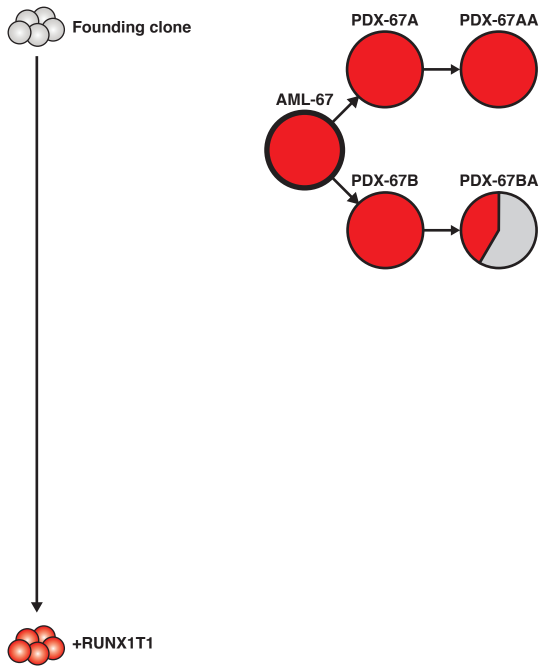

b

LOSS

Gene	AML-20	PDX-20A	PDX-20B	PDX-20C	PDX-20D	PDX-20E
PDS5A	96	94	77	77	98	76
ROS1	92	100	62	74	88	92
**CBFB/MYH11**	+	+	+	+	+	+
CDHR2	100	88	59	81	37	64
CFAP61	100	100	43	84	29	96
KIAA1217	97	68	56	95	56	100
TTN	89	92	62	75	63	83
TAF2	80	76	54	59	35	84
BCKDHB	73	76	66	64	63	89
**NRAS**	67	58	47	71	24	71

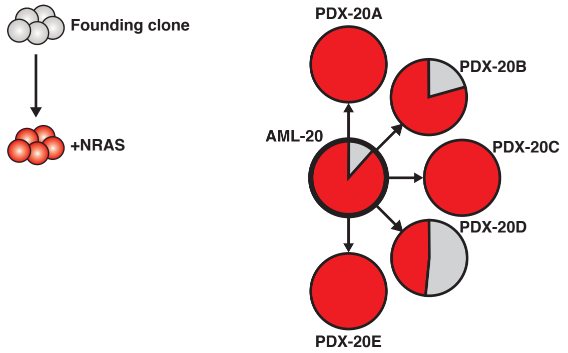

c

LOSS

Gene	AML-26	PDX-26A	PDX-26AA	PDX-26B	PDX-26BB	PDX-26C
MYRF	100	100	100	83	82	100
BPIFB3	100	78	100	81	88	80
**DNMT3A**	100	81	71	100	90	100
LPCAT2	100	99	63	100	85	87
LARS	100	94	100	100	93	100
WDR36	100	79	90	88	89	100
MYO5B	100	85	100	98	100	100
DYSF	100	89	100	100	100	71
CKMT1B	100	84	88	82	93	94
TMEM44	100	75	100	100	96	100
**IDH2**	99	100	88	91	91	75
TRAFD1	98	100	99	95	94	80
KCNB1	97	94	100	79	80	94
CLASP1	94	83	83	100	98	92
SOS1	94	100	100	100	100	96
COL4A5	91	96	89	96	86	90
DLEC1	90	82	93	97	96	100
ERCC2	87	96	100	79	100	88
MAMSTR	84	93	95	100	100	90
ANKRD30B	82	89	92	77	99	80
**DNMT3A**	82	96	100	67	100	64
SEMA5A	76	97	83	96	92	95
DPY19L2P4	73	71	84	96	91	100
ACAN	64	100	100	89	78	100
**NPM1**	53	69	76	65	62	69
GLTSCR1	0	9	2	5	52	4
ZNF235	0	1	16	10	39	4
MMP15	0	2	12	7	29	10
MASP2	100	100	89	96	56	87
FASN	94	100	40	85	56	91
CDHR3	81	88	61	81	65	82
**del(19)(p13)**	81	90	67	85	52	84
PCLO	79	92	87	92	64	80
SULT2B1	78	100	89	75	60	56
PRAMEF1	71	46	93	82	70	91

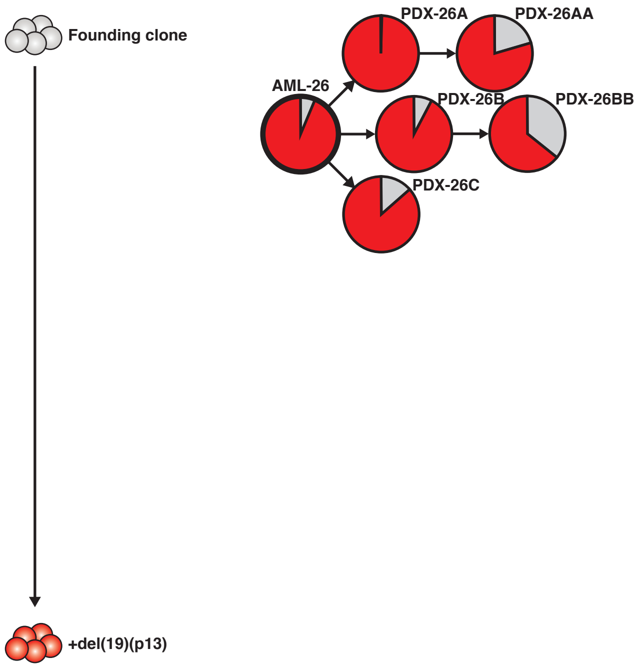

Supplementary Figure 2. Additional xenografts with the Loss pattern. (a-c) Three additional cases with the Loss pattern of clonal dynamics.

a

EXPANSION

	AML-16	PDX-16A	PDX-16B	PDX-16BA	PDX-16BB
THADA	100	100	100	100	100
MYT1L	100	88	78	100	100
**FLT3**	98	85	100	93	100
**STAG2**	93	100	100	100	100
GGT8P	92	78	88	100	90
IGSF9B	92	100	90	84	74
**TET2**	86	100	93	100	100
EVPL	85	100	92	100	83
MYO3A	85	100	91	70	97
ABCA4	80	100	100	79	100
ZFYVE9	79	61	60	83	97
NRP2	67	80	85	100	78
WDR17	58	100	98	99	100
HMCN2	58	94	94	85	88
**NPM1**	47	47	62	57	63
MANF	21	61	100	98	78
**LOH(4)(q21-35)**	0	94	92	100	100
PCDHB7	0	0	10	77	15
**del(2)(q36)**	0	0	0	0	89
SCRN1	0	0	0	74	7
**ATRX**	0	0	0	72	1

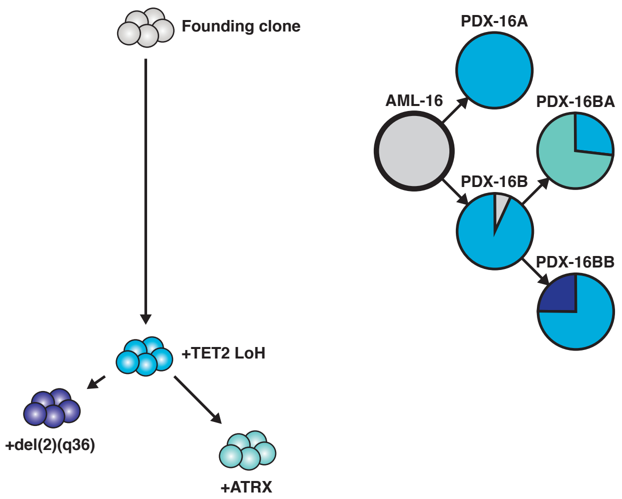

b

EXPANSION

	AML-17	PDX-17A	PDX-17AB	PDX-17AC
PCDH11Y	86	100	100	100
ACOT1	73	83	97	100
LOC101928697	100	74	100	100
WBSCR17	100	83	100	100
PSMB7	100	71	90	85
ADGRV1	96	99	94	74
MMP16	96	45	52	89
MEGF10	95	91	95	100
**DNMT3A**	92	100	96	81
KIAA0226	91	97	96	99
FLT3	89	100	100	84
ZNF366	89	79	89	90
DSC3	89	85	99	89
HMCN1	85	90	100	95
PEPD	82	85	100	100
PEX16	81	85	94	100
AKAP13	79	85	63	92
SPTA1	76	69	100	100
EPHA7	73	99	97	83
DNAH3	73	100	100	100
PATL1	70	89	92	100
**GATA2**	59	64	72	100
NMUR2	51	80	74	99
**NPM1**	50	58	74	80
KRT28	49	63	100	90
KIAA0922	87	38	1	0
**NRAS**	82	30	0	0
KIF2B	74	27	0	0
**WT1**	0	52	88	100
WDR62	1	50	87	99
CCT6B	0	0	100	2
PAPPA	0	0	89	0
PDE2A	0	0	3	96

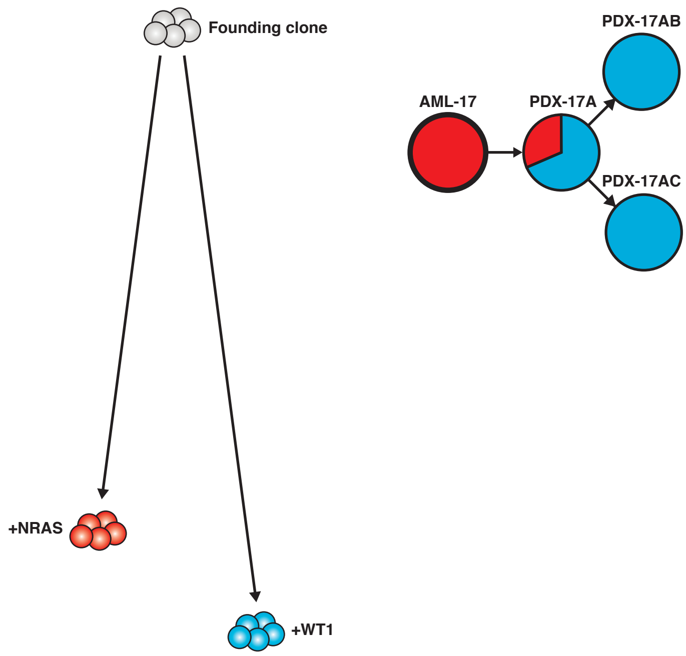

c

EXPANSION

	AML-21	PDX-21A	PDX-21AA	PDX-21AC	PDX-21D	PDX-21E	PDX-21F	PDX-21G	PDX-21H	PDX-21I
EDAR	100	94	100	91	100	92	62	85	92	74
ANXA2P2	100	91	89	100	100	100	100	98	95	89
TTN	100	100	90	92	86	99	95	100	89	94
TUBB3	90	94	96	79	99	100	100	100	100	100
**STAG2**	89	97	89	95	100	100	94	98	99	98
SH2B3	87	100	86	97	93	99	80	100	88	92
**IDH2**	84	100	90	99	77	96	96	90	91	86
MPP4	79	86	100	82	100	87	100	92	100	100
HNF1A	77	87	100	90	93	100	100	91	92	100
**SRSF2**	72	100	87	100	86	NA	NA	100	90	98
SERPINB4	71	100	91	84	100	91	100	95	100	94
TGM4	67	100	78	100	100	101	92	100	95	97
**BCOR**	78	0	0	0	0	0	0	3	0	0
USP9X	38	0	0	0	0	0	2	0	0	1
SARS	25	100	81	100	84	100	85	100	100	90
GRIA4	18	100	100	91	100	88	99	100	83	82
DRD5	16	87	91	65	67	100	79	85	100	100
MMP13	12	100	77	90	91	91	81	88	100	93
**NRAS**	6	94	94	98	89	89	74	79	80	100
TGFBR3	4	75	80	84	72	73	54	100	72	95
PROX1	4	97	91	100	99	100	98	86	93	71
TANGO6	4	69	32	57	36	84	57	74	95	65
DNM3	0	0	0	0	0	0	80	0	1	0
NOTCH3	0	0	0	0	0	0	3	74	0	0
DHRS7C	0	0	0	0	0	0	0	0	41	0
ZNF322	0	0	0	0	0	0	0	0	31	0
SMG1	4	0	0	0	3	1	0	2	1	84
**LOH(11)(q22-25)**	0	0	79	74	0	0	0	0	0	0
**+11(p11-15)**	0	0	0	0	96	0	0	0	0	0
**del(6)(p24-25)**	0	0	0	0	67	0	0	0	0	0
OR7D4	0	0	0	0	54	0	0	0	0	0

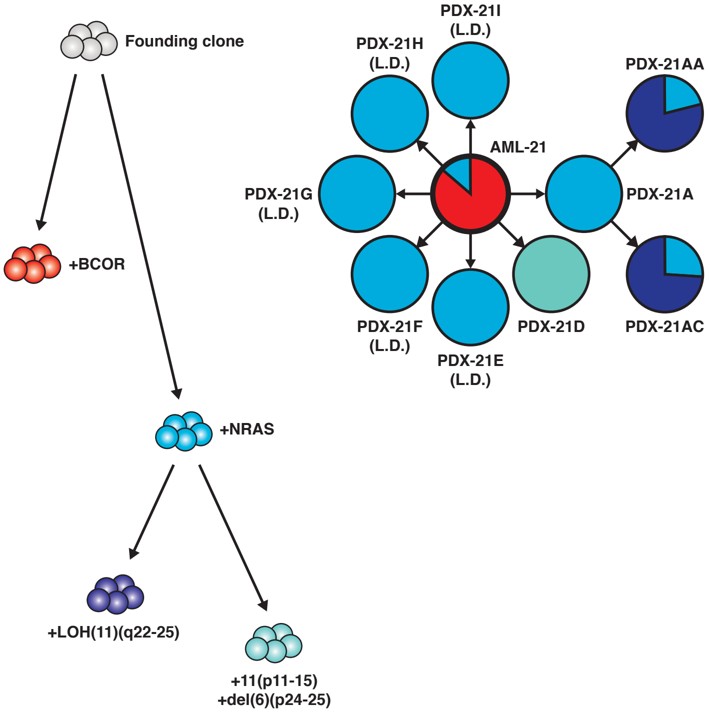

d

EXPANSION

	AML-74	PDX-74A	PDX-74AA	PDX-74AB
ANKRD44	92	96	100	100
ZNF45	78	99	75	99
AKAP8	75	78	76	94
MIR5684	75	67	94	88
LHFPL3	74	75	100	100
XDH	73	100	77	92
TDRD5	65	100	100	95
APLN	64	100	100	97
**IDH1**	62	66	91	83
ACE	62	100	100	100
TJP1	61	83	100	94
SSPO	59	68	87	59
COL6A3	56	82	100	82
SLC35E3	56	63	92	100
KCNH1	54	99	75	81
CRELD2	54	100	100	100
CNTNAP5	54	98	97	93
CHD9	53	88	100	90
SCAMP2	53	75	80	100
TUSC3	50	100	100	80
STX11	41	0	0	0
**del(12)(p11-13)**	9	92	100	100
DIP2B	8	79	100	86
CTNNA2	5	100	53	100
NUPR1L	2	59	84	78
RPL5	0	28	92	100
SOST	0	0	62	0
ZNF503-AS2	0	0	0	88

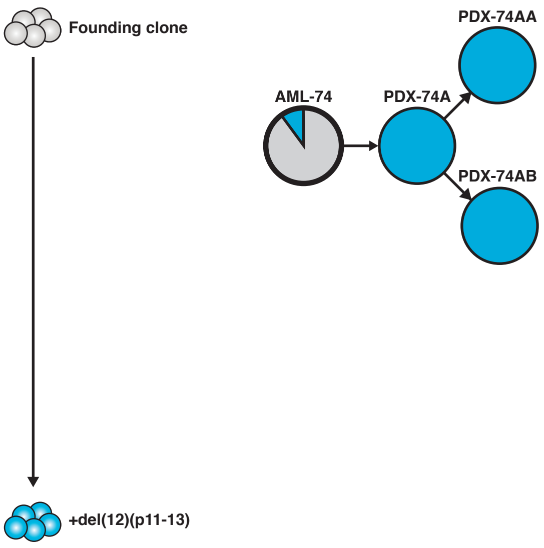

e

EXPANSION

	AML-37	PDX-37C	PDX-37D	PDX-37E
CCDC26	80	100	99	100
**TP53**	76	100	95	97
DNTTIP1	67	100	97	89
IL12A-AS1	66	100	100	95
SPR	66	100	100	100
SLC25A26	63	69	100	86
DRD2	62	100	95	76
SPG20	60	88	94	79
ERBB4	60	100	94	100
**i(22)**	60	100	98	100
**i(8)**	59	94	90	97
SEC61B	58	93	94	85
CRAMP1L	58	96	92	90
KIF21A	56	97	99	86
**FLT3**	55	101	96	88
**LOH(17)(p12-13)**	51	100	99	96
CNGB1	35	89	92	72
**+6**	17	76	76	100
**CDKN2A**	16	71	55	73
**i(18)**	7	49	52	90

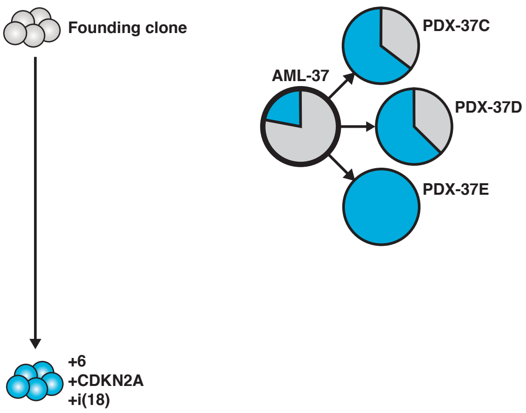

f

EXPANSION

	AML-33	PDX-33B	PDX-33BA	PDX-33C	PDX-33CA
SPTBN2	98	100	100	100	78
MRPL52	96	100	100	82	100
ANAPC2	96	100	79	100	73
ZNF644	91	100	100	100	100
NPHP4	90	100	98	100	88
ADGRV1	90	100	94	100	88
BMPR1A	89	85	95	94	89
EPHA3	87	97	100	92	100
**IDH1**	87	86	53	100	70
PKD1L2	87	90	96	100	89
C15orf41	85	62	80	83	100
CLSTN3	84	75	100	82	100
MGAM	80	100	72	95	100
TYRO3	78	100	100	100	100
SLC6A19	77	94	91	100	84
**DNMT3A**	77	95	99	100	100
TTYH1	77	100	98	100	100
TTN	76	79	19	78	48
IARS	74	85	93	100	100
LMAN1	52	74	24	78	43
**NPM1**	49	74	34	68	51
**FLT3-ITD**	+	+	+	+	+
**LOH(13)**	35	96	97	96	100
ZNF845	4	1	49	0	35
TFAM	0	0	48	0	50
PLBD1	0	3	64	0	14
MAGEA4	0	0	56	0	24

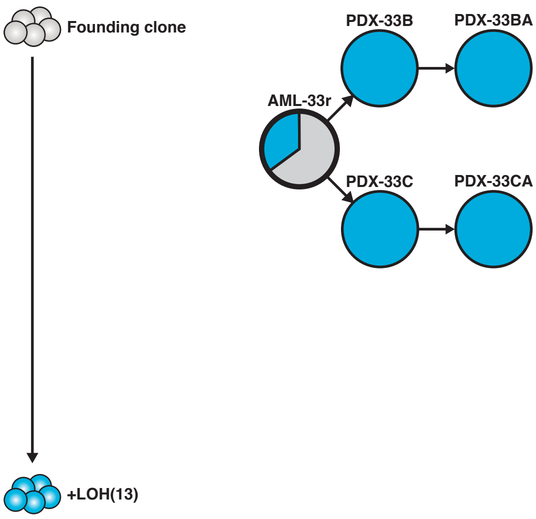

g

EXPANSION

	AML-83	PDX-83A	PDX-83B	PDX-83BA	PDX-83BC	PDX-83C
MCM4	100	100	96	100	81	100
CPNE4	100	100	100	91	100	100
PTPRB	100	100	100	100	100	99
GOT2	97	58	53	77	90	100
OSBPL8	97	100	100	97	100	100
RIMS2	95	92	100	100	90	94
KIF14	94	100	85	100	100	95
ITGBL1	92	80	81	100	100	88
**+11(q23-25)**	92	100	100	100	100	100
RNF217-AS1	91	89	82	100	82	84
CCDC67	89	95	76	65	70	100
SNORD80	88	78	97	100	100	100
**TP53**	88	98	100	100	97	100
IMPG2	87	86	100	92	100	110
**NF1**	87	100	100	97	99	98
SURF1	86	78	100	100	82	100
HAL	84	93	91	100	99	94
MYO6	84	100	98	95	99	100
SNAP91	83	50	87	100	89	100
**-5q**	83	100	100	100	100	100
**del(10)(p13-15)**	83	100	100	100	100	100
**del(20)(q12-13)**	83	100	100	100	100	100
**-16p**	83	100	100	100	100	100
ERBB3	82	100	100	92	99	100
**LOH(17)**	82	100	100	100	100	100
PRPF8	82	97	100	95	100	98
UNC5A	81	100	100	100	100	100
CLTCL1	80	98	97	83	85	100
**TET2**	80	75	92	78	76	87
**del(9)(p23-q22)**	79	100	100	100	100	100
FBN2	79	98	97	100	100	99
SEP15	79	100	100	100	100	100
**-7q**	78	96	95	96	97	95
C19orf80	77	65	100	83	100	100
PPFIA2	76	97	100	100	100	100
DSG1	76	100	100	93	91	100
MIA-RAB4B	75	95	80	88	96	94
SPATA5	74	84	99	65	100	98
APOB	73	77	87	100	85	84
FGF20	66	99	94	99	90	100
**del(10)(p11-12)**	64	100	100	100	100	100
FAM208B	63	100	100	100	100	98
ARHGEF33	63	65	100	81	79	68
ERBB2IP	58	85	86	90	76	89
SULT1B1	50	61	100	100	90	100
C2orf71	50	80	92	60	74	57
A1CF	79	0	55	0	0	72
HTN1	1	0	21	0	0	52
MVK	0	0	12	0	0	65
**del(13)(q13-31)**	0	0	0	0	0	41
**del(14)(q12-31)**	0	0	0	0	0	39
**del(10)(q11-21)**	17	73	65	97	98	32
CTNNB1	33	99	39	0	0	23
FMN2	25	79	30	0	3	22
NFATC4	18	100	51	0	0	22
GNAI2	7	83	19	0	0	11
MYH1	5	92	12	0	0	19
ZNF385D	4	100	16	3	4	27
DSP	3	100	30	0	0	13
CDH6	3	89	58	4	0	14
PLCE1	0	29	0	0	1	0
WDR33	31	0	65	100	98	25
CHD4	10	0	41	93	80	14
KIF5B	5	0	47	78	96	18
HJURP	2	0	73	30	90	6
LOC644145	0	0	66	54	100	0
NDUFS5	0	0	3	65	5	0
**del(10)(q21-23)**	0	86	0	0	0	0
PSIP1	0	22	0	0	0	0

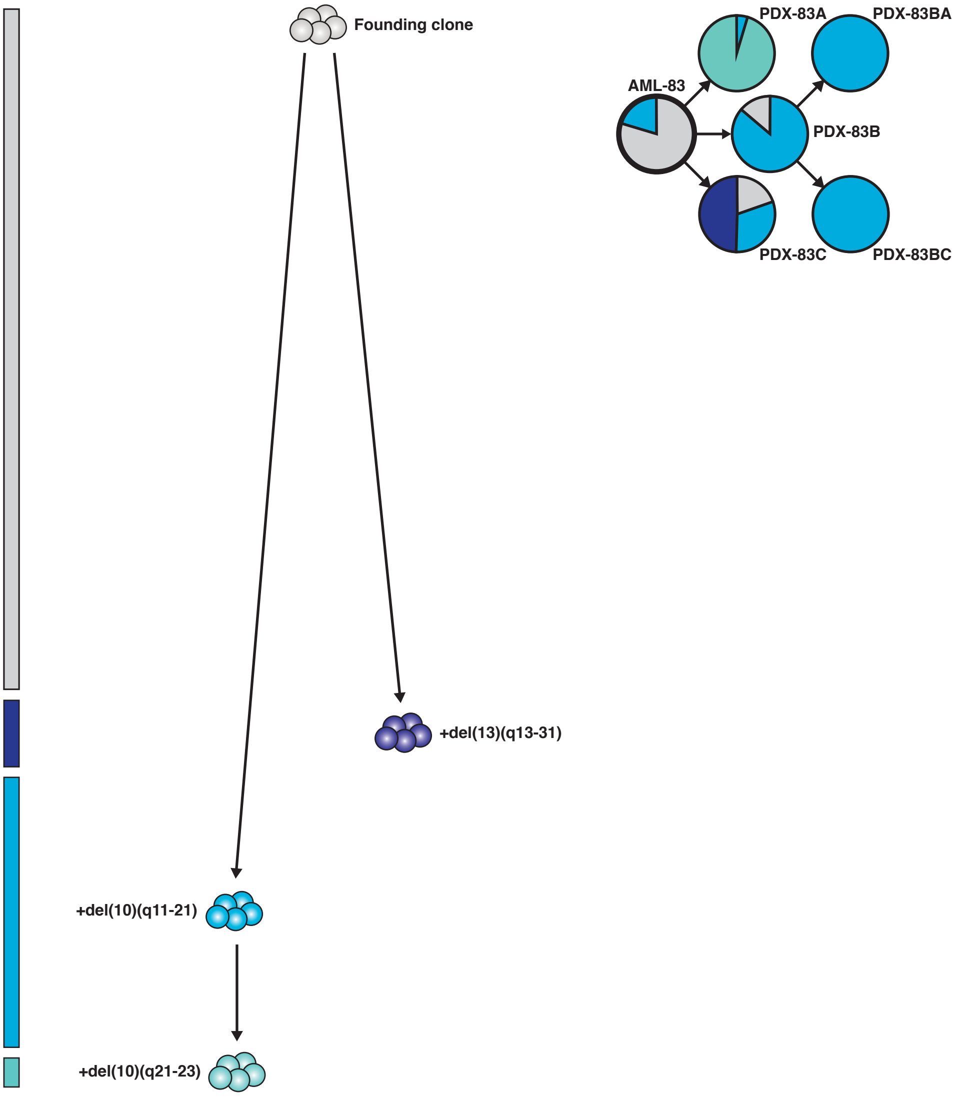

h

EXPANSION

	AML-107	PDX-107A	PDX-107AA
SCAF8	51	100	77
NECAB1	49	83	100
ADCY2	49	100	100
**del(7)(q33-35)**	45	100	100
**-5q**	42	100	100
**+11(q22-25)**	42	100	100
**-17(p12-13)**	41	100	100
ODF2	38	82	96
**TP53**	37	96	94
**-12(p12-13)**	37	100	100
BPIFB2	37	71	96
ZCCHC12	36	79	88
NAPB	34	100	77
TBX22	30	70	82
SALL3	27	100	100
DIAPH3	25	100	100
**-15q**	21	100	100
TYR	30	1	1
PHF3	10	96	73
**CBL**	7	100	100
CTSS	1	100	88
**-19(p12-q13)**	0	100	100
PCDH17	0	98	37
SLC24A1	0	75	39
TPTE	1	31	28

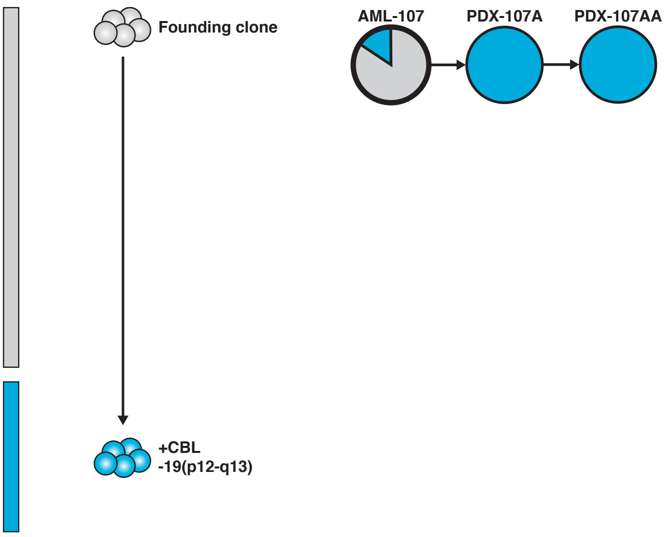

i

EXPANSION

	AML-54	PDX-54A
FTCDNL1	100	87
ARHGAP22	100	78
GYG2P1	93	100
FAF2	90	100
**RUNX1**	88	100
SMG5	87	83
CNOT7	86	75
TTC7B	84	93
C17orf70	84	100
**-7**	83	100
TNP2	83	93
ATM	81	91
ZNF385D	79	97
PCCA	79	100
SKI	79	88
STEAP1B	78	100
PIP4K2A	77	93
UBR4	77	100
PAX3	77	95
PZP	76	99
PROCA1	76	100
CFTR	75	100
RPL26L1	74	100
TUSC3	72	96
TEX9	72	86
PLTP	71	92
KIAA1804	70	90
SEPT4-AS1	69	100
KDM4B	67	81
CYP4A11	67	100
PIP5K1C	66	83
KSR2	65	100
SOWAHB	65	89
MFSD8	64	100
GRM4	63	99
ROCK1	61	100
UNC45B	61	100
KCTD16	59	51
TTN	56	66
ECI2	55	89
KIF2B	39	99
**EZH2**	27	67
**+8**	14	100
**LOH(11)(p11-15)**	0	91
**BCOR**	27	4
DNM2	26	0
**ETV6**	17	0
LAMA1	15	0
AGXT2	10	0
**CEBPA**	5	0

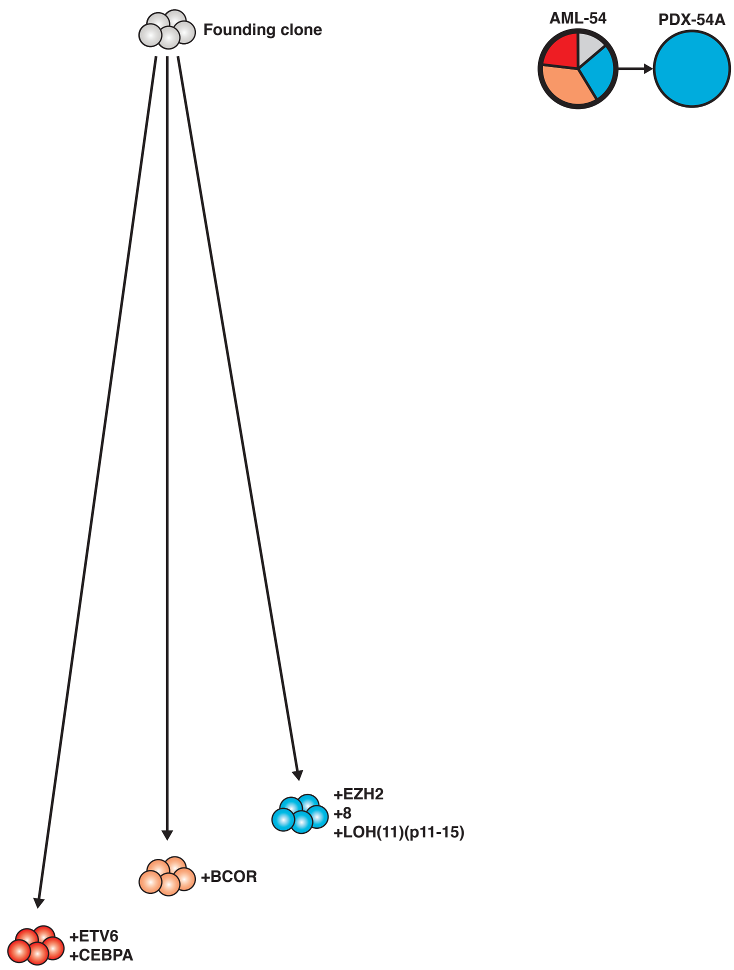

j

EXPANSION

	AML-48	PDX-48A
MIR548AI	100	100
NBPF6	100	84
GCK	100	83
**TP53**	98	89
CNTN4	97	79
PNPLA1	97	100
DOCK8	97	100
ZFYVE9	96	85
BBS9	94	93
MIR1973	94	92
CYP19A1	92	98
**-20(q11-13)**	90	100
MYOC	89	100
CREBBP	89	75
**-5(q14-34)**	89	100
THBS1	89	100
SMG1	88	88
DNAH5	88	100
**-8(p12-23)**	87	100
**TP53**	86	100
ATP6V1B1	85	78
IL4	85	100
**iso(16)**	85	100
**-18**	85	100
**-12(p12-13)**	85	100
SHISA8	84	98
**i(22)**	84	100
FOXA1	83	93
RBBP8NL	83	79
TKTL1	83	94
RPS6KC1	82	100
PLEKHH1	77	91
PHACTR4	77	78
WISP3	76	80
NRG3	74	69
PDLIM3	73	100
HYOU1	71	85
USP17L10	50	71
ERAP2	14	89
**-17(p11-q12)**	0	78
**-17(q24)**	0	77
CHST5	0	72
SLC28A3	2	24
MTHFD1	0	23
ICA1L	0	16
**i(11)**	53	0

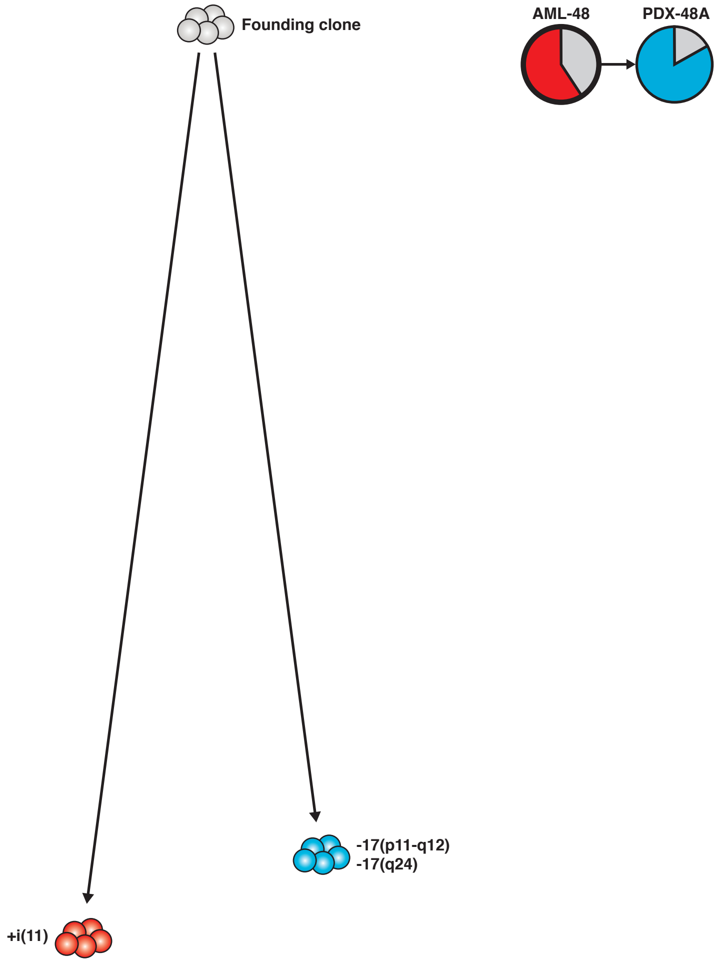

Supplementary Figure 3. Additional xenografts with the Expansion pattern. (a-j) Ten additional cases with the Expansion pattern of clonal dynamics.

BURST

	AML-7	PDX-7A	PDX-7AA	PDX-7B
ZFP90	98	88	100	100
F8	94	94	100	100
TSEN34	94	100	100	100
MKRN3	90	74	98	94
**DNMT3A**	88	74	88	100
MKRN3	86	96	100	100
TRIM42	86	100	84	100
SLC36A4	86	98	100	100
BCL11A	76	84	84	96
ALPK2	72	98	100	90
ABCC8	60	100	64	100
SPEG	60	96	52	84
OR2M3	58	64	34	48
**NPM1**	44	70	56	58
FAT4	44	10	0	0
**WT1**	44	10	14	0
RIT1	44	10	6	0
COL6A3	34	22	0	0
APOB	28	8	0	0
**WT1**	26	8	6	0
TTN	18	58	46	60
**IDH2**	18	70	68	50
WRN	18	68	84	90
ROBO1	14	36	80	84
JAKMIP3	12	40	64	100
NFE2	0	6	70	28
**NF1**	0	2	58	30
TLR7	0	0	42	0
**FLT3**	12	0	14	74
**PTPN11**	2	30	0	4

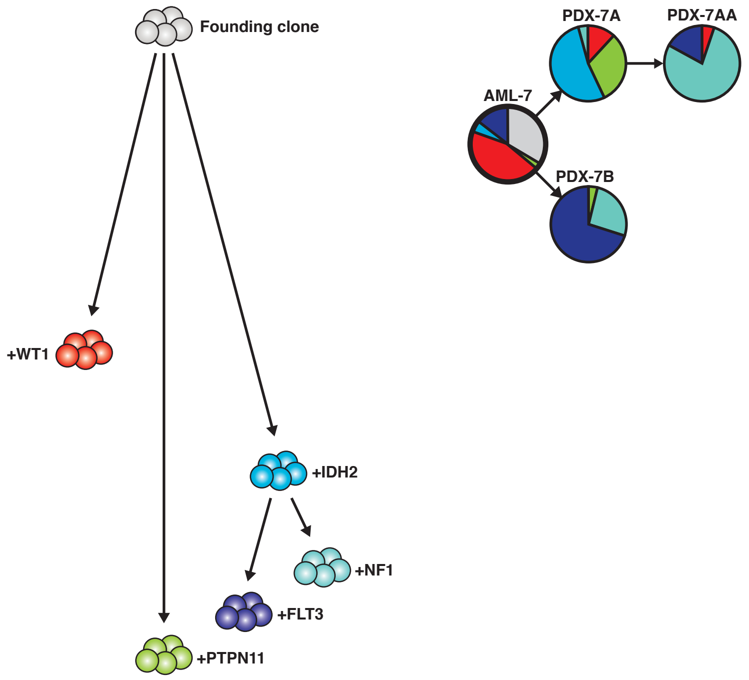

Supplementary Figure 4. Additional xenografts with the Burst pattern. One additional case with the Burst pattern of clonal dynamics.

a

Gene	AML-7	PDX-7A	PDX-7AA	PDX-7B	Relapse
ZFP90	98	88	100	100	8
F8	94	94	100	100	22
TSEN34	94	100	100	100	40
MKRN3	90	74	98	94	24
**DNMT3A**	88	74	88	100	24
MKRN3	86	96	100	100	16
TRIM42	86	100	84	100	14
SLC36A4	86	98	100	100	20
BCL11A	76	84	84	96	28
ALPK2	72	98	100	90	12
ABCC8	60	100	64	100	54
SPEG	60	96	52	84	14
OR2M3	58	64	34	48	28
**NPM1**	44	70	56	58	8
FAT4	44	10	0	0	0
**WT1**	44	10	14	0	0
RIT1	44	10	6	0	0
COL6A3	34	22	0	0	0
APOB	28	8	0	0	0
**WT1**	26	8	6	0	0
TTN	18	58	46	60	20
**IDH2**	18	70	68	50	26
WRN	18	68	84	90	20
ROBO1	14	36	80	84	8
JAKMIP3	12	40	64	100	12
NFE2	0	6	70	28	20
**NF1**	0	2	58	30	4
TLR7	0	0	42	0	0
**FLT3**	12	0	14	74	0
**PTPN11**	2	30	0	4	0

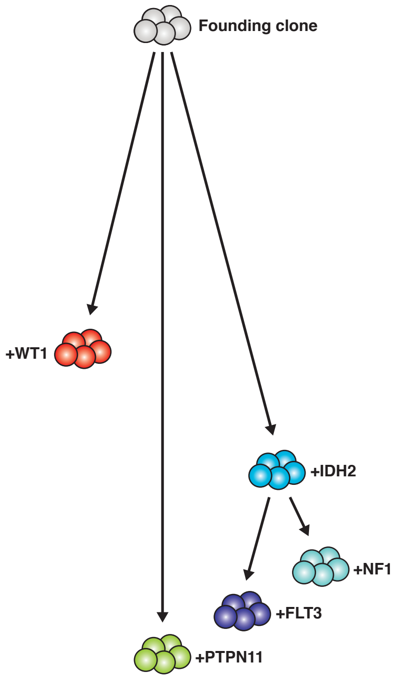

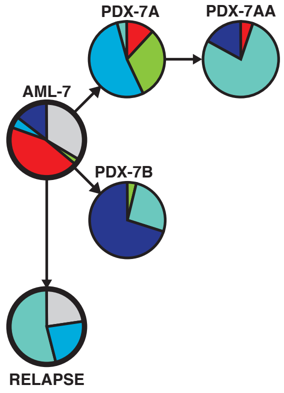

b

Gene	AML-11	PDX-11D	PDX-11E	Relapse
ANKRD30B	100	92	100	78
F3	100	92	100	81
**U2AF1**	90	67	93	100
**DNMT3A**	80	100	100	100
**TET2**	80	90	91	82
C9orf152	68	96	100	87
**TET2**	67	68	85	100
USP42	57	94	82	92
FCGR3B	40	45	93	66
ABCA1	62	91	20	0
MAGEB18	33	100	20	0
MIR1268A	79	95	0	0
SRCAP	59	90	0	0
MGAT5	57	77	0	1
PDS5B	57	100	0	0
**NPM1**	43	54	0	0
ARHGEF28	0	100	0	3
STK25	0	89	0	0
OR10G8	88	0	0	0
ATXN1	73	0	0	0
**LOH(9)(p12-24)**	71	0	0	0
SSX8	69	0	0	1
**CDKN2A**	64	0	0	0
OR10K2	63	0	0	0
REG1A	61	1	4	0
**KRAS**	59	0	0	0
HIST2H2AC	55	0	0	0
C10orf12	49	0	0	0
ULK1	0	0	0	100
NUP205	0	0	0	97
OPA3	0	0	0	94
TMEM214	0	0	0	91
**del(11)(q23)**	0	0	0	86
ABCC12	0	0	0	81
PHEX	0	0	0	80
CD244	0	4	6	72
HTR2C	0	0	0	65
**RUNX1**	0	0	0	47
ABCC9	0	1	0	42

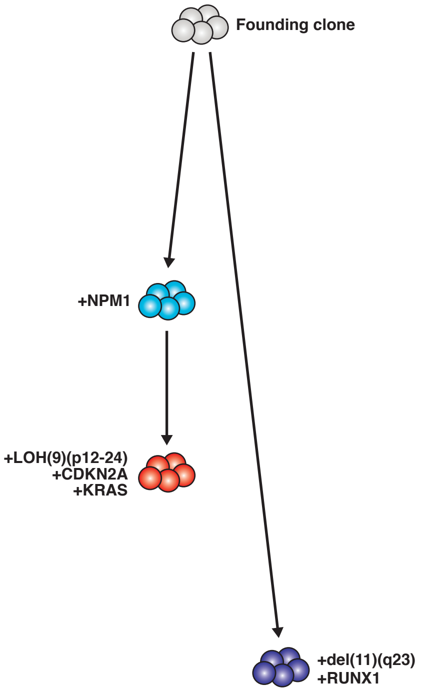

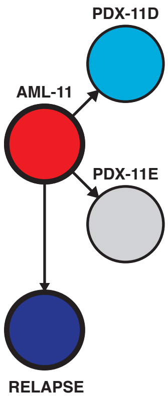

c

Gene	AML-20	PDX-20A	PDX-20B	PDX-20C	PDX-20D	PDX-20E	Relapse
PDS5A	96	94	77	77	98	76	6
ROS1	92	100	62	74	88	92	16
**CBFB/MYH11**	+	+	+	+	+	+	+
CDHR2	100	88	59	81	37	64	2
CFAP61	100	100	43	84	29	96	5
KIAA1217	97	68	56	95	56	100	5
TTN	89	92	62	75	63	83	4
TAF2	80	76	54	59	35	84	6
BCKDHB	73	76	66	64	63	89	3
**NRAS**	67	58	47	71	24	71	0

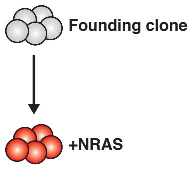

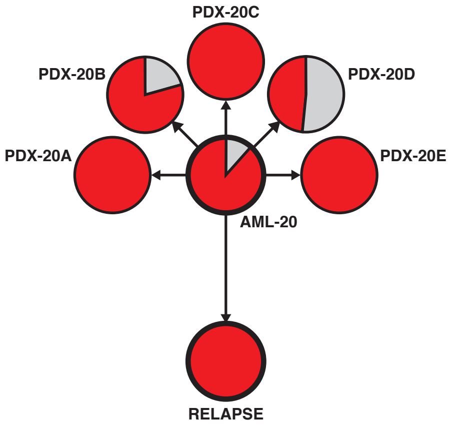

d

Gene	AML-54	PDX-54A	Relapse
FTCDNL1	100	87	74
ARHGAP22	100	78	86
GYG2P1	93	100	72
FAF2	90	100	77
**RUNX1**	88	100	71
SMG5	87	83	70
CNOT7	86	75	83
TTC7B	84	93	72
C17orf70	84	100	76
**-7**	83	100	65
TNP2	83	93	86
ATM	81	91	63
ZNF385D	79	97	60
PCCA	79	100	65
SKI	79	88	73
STEAP1B	78	100	66
PIP4K2A	77	93	71
UBR4	77	100	66
PAX3	77	95	74
PZP	76	99	78
PROCA1	76	100	73
CFTR	75	100	77
RPL26L1	74	100	62
TUSC3	72	96	83
TEX9	72	86	57
PLTP	71	92	70
KIAA1804	70	90	78
SEPT4-AS1	69	100	66
KDM4B	67	81	72
CYP4A11	67	100	59
PIP5K1C	66	83	68
KSR2	65	100	44
SOWAHB	65	89	69
MFSD8	64	100	66
GRM4	63	99	70
ROCK1	61	100	82
UNC45B	61	100	70
KCTD16	59	51	78
TTN	56	66	62
ECI2	55	89	53
KIF2B	39	99	53
**EZH2**	27	67	0
**+8**	14	100	0
**LOH(11)(p11-15)**	0	91	0
**BCOR**	27	4	0
DNM2	26	0	0
**ETV6**	17	0	76
LAMA1	15	0	71
AGXT2	10	0	61
**CEBPA**	5	0	57
**KRAS**	0	0	66
MAGEB3	0	0	71
LIFR	0	0	73

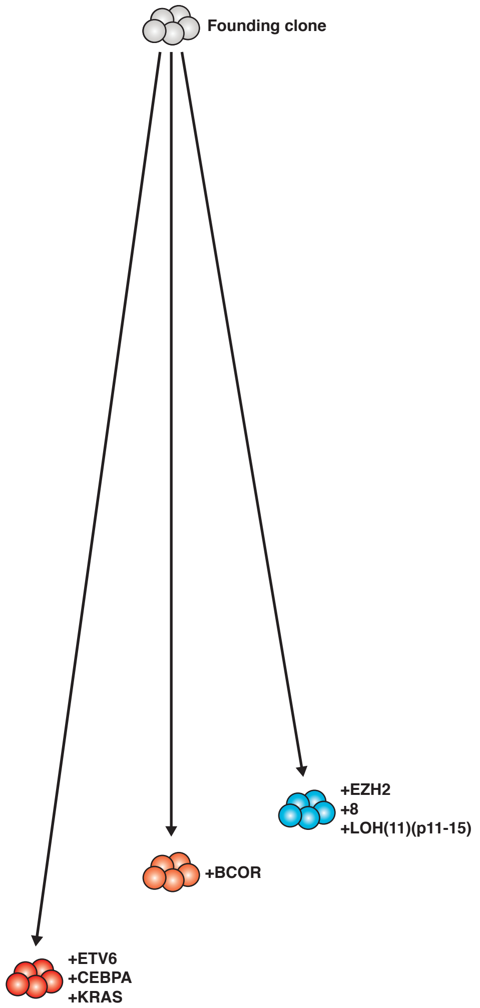

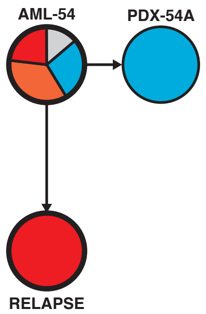

e

Gene	AML-33	PDX-33B	PDX-33BA	PDX-33C	PDX-33CA	Relapse
SPTBN2	98	100	100	100	78	41
MRPL52	96	100	100	82	100	25
ANAPC2	96	100	79	100	73	32
ZNF644	91	100	100	100	100	30
NPHP4	90	100	98	100	88	32
ADGRV1	90	100	94	100	88	33
BMPR1A	89	85	95	94	89	52
EPHA3	87	97	100	92	100	31
**IDH1**	87	86	53	100	70	18
PKD1L2	87	90	96	100	89	17
C15orf41	85	62	80	83	100	26
CLSTN3	84	75	100	82	100	16
MGAM	80	100	72	95	100	59
TYRO3	78	100	100	100	100	43
SLC6A19	77	94	91	100	84	37
**DNMT3A**	77	95	99	100	100	37
TTYH1	77	100	98	100	100	29
TTN	76	79	19	78	48	27
IARS	74	85	93	100	100	38
LMAN1	52	74	24	78	43	25
**NPM1**	49	74	34	68	51	18
**FLT3-ITD**	+	+	+	+	+	+
**LOH(13)**	35	96	97	96	100	0
ZNF845	4	1	49	0	35	0
TFAM	0	0	48	0	50	0
PLBD1	0	3	64	0	14	0
MAGEA4	0	0	56	0	24	0

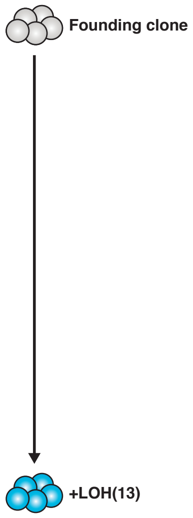

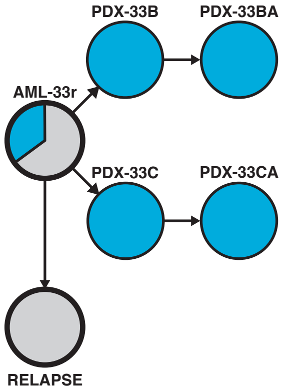

f

	AML-9	PDX-9B	PDX-9C	Relapse
PRKCG	78	71	81	78
FARS2	73	75	95	59
NUDT6	70	87	84	75
TMEM35	69	90	97	75
**DNMT3A**	69	98	100	81
**TET2**	69	95	100	74
MAPK11	67	100	100	73
FBN2	67	100	100	77
ENPP3	67	85	92	76
PCDHGB1	65	97	99	73
ECHDC1	64	93	95	74
OR5K2	60	100	91	68
ANK2	59	84	96	63
APC	57	100	100	80
CACNA1B	57	99	84	73
**TET2**	55	89	97	58
DVL1	53	100	74	62
CCBL2	43	71	94	65
GALNT6	35	100	98	69
**NPM1**	32	87	84	44
GPR55	32	94	91	74
ANKRD20A4	27	87	81	34
KMT2C	2	56	24	8
FAM72C	1	39	42	14
PLEKHG4B	0	0	0	89
GSAP	0	0	0	68
PLEC	0	0	0	62

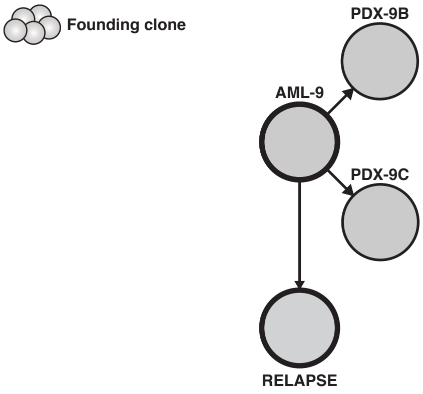

g

	AML-79	PDX-79A	PDX-79B	PDX-79C	Relapse
ZIC2	100	100	96	100	44
ITIH3	97	96	100	94	15
GADL1	97	98	100	86	40
IL31RA	97	100	100	100	24
AGXT	96	82	100	73	38
CCDC54	96	100	100	100	31
DSCAML1	93	84	91	66	36
CCDC168	90	100	88	100	36
ENTPD4	89	78	100	96	40
**IDH2**	89	75	91	82	36
RYR2	88	88	100	100	42
ESPNL	87	88	85	74	26
IRF5	86	99	100	46	33
SORCS2	85	83	92	82	33
KRT39	84	90	84	71	32
NPAP1	83	91	91	74	35
OR4B1	80	83	94	58	34
TGS1	78	94	86	76	19
XDH	77	86	80	85	30
MYO5C	75	100	100	77	25
**NPM1**	74	83	62	85	18
SLC6A1	71	71	84	74	27
COL15A1	67	86	98	100	25
SIGLEC1	64	85	75	100	32
C14orf180	1	0	0	0	29
SNORD116-25	0	0	0	0	44
ADAM29	88	85	100	78	1
WASH2P	83	82	82	71	0
NHEJ1	74	91	89	69	0
**FLT3-ITD**	68	72	86	84	0

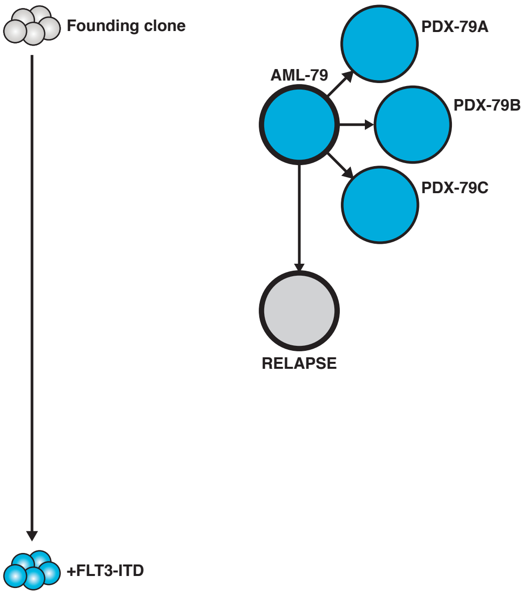

Supplementary Figure 5. AML Xenografts can reveal relapse clones but not predict clonal evolution from diagnosis to relapse. AML xenografts identified the relapsing clone in the diagnostic sample from AML-7 (a) and partially recapitulated the clonal dynamics from diagnosis to relapse in AML-11 (b) but did not predict the evolution from diagnosis to relapse in AML-20 (c), AML-54 (d), AML-33 (e) and AML-79 (g). AML-9 (f) contained the same clone at diagnosis and relapse. The presence of FLT3-ITD in AML-33 and corresponding xenografts is denoted by "+", as the rearrangement in this case is detectable but not quantifiable by WES.

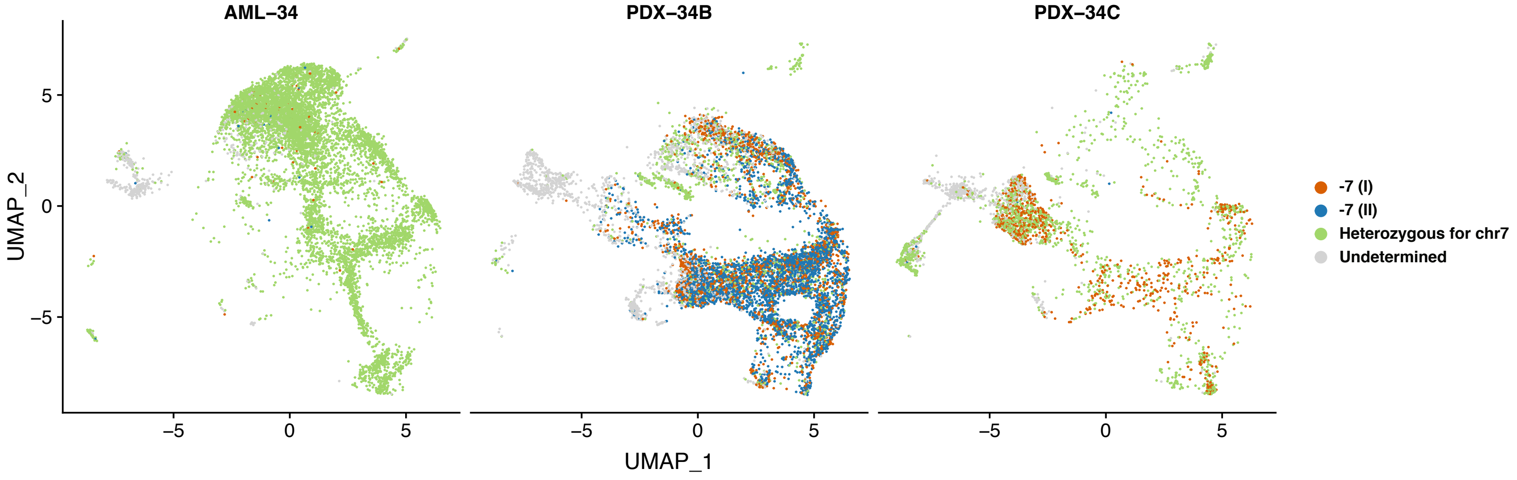

Supplementary Figure 6. Validation of clonal dynamics by single cell sequencing. Identification of monosomy 7 by single cell RNA sequencing through detection of SNVs in expressed transcripts from chromosome 7, confirming the expansion of clones with losses of either allele of chromosome 7 in two independent xenografts.

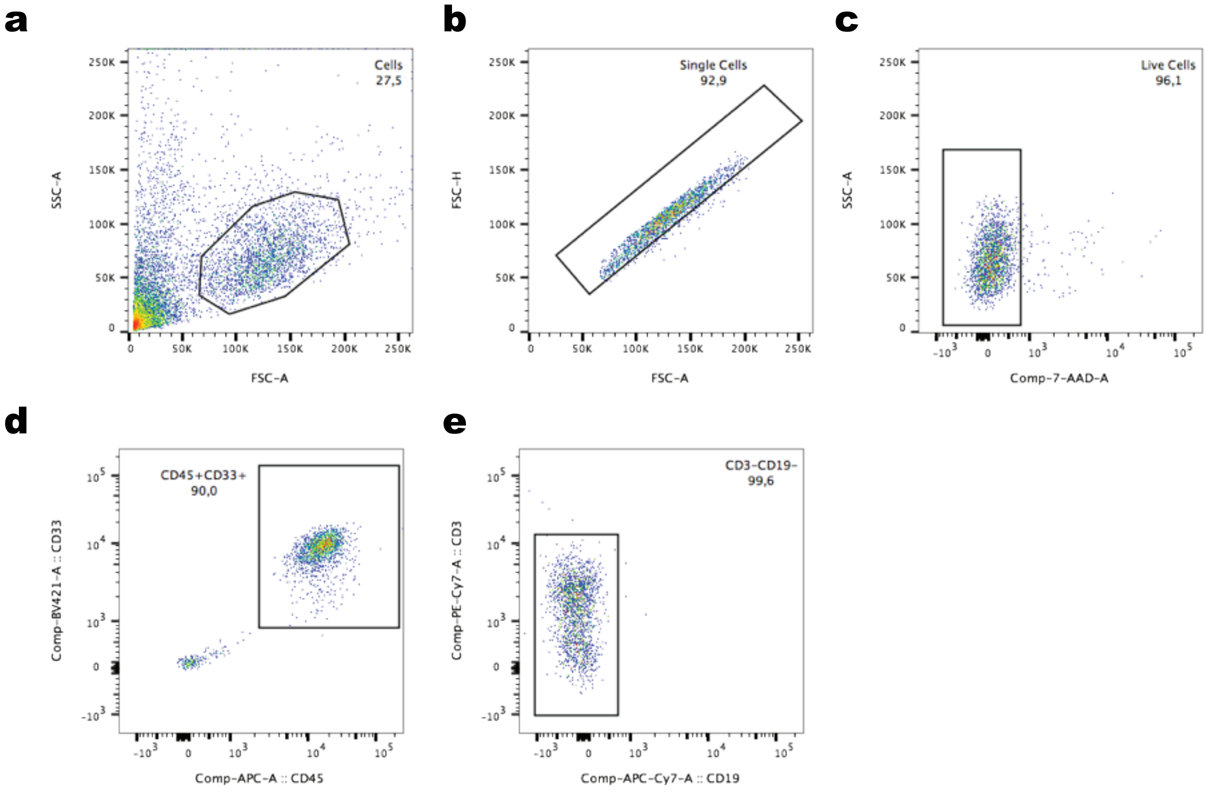

Supplementary Figure 7. Gating strategy for flow cytometry. Leukemic engraftment was assessed as the percentage of hCD45+CD33+CD3-CD19- cells within the live cell compartment through sequential gating from (a) to (e).

Supplementary Methods

Algorithmic clone calling

Manual clone calling was validated by clone calling using the PyClone software.[1] The PyClone results confirmed the original clone calling, except in three instances where the algorithm produced less credible clonal structures, thus validating the manual approach. First, in four samples with insertions in *NPM1* (AML-7, AML16, AML-26 and AML-28), PyClone separated the *NPM1* alteration from the other founding clone mutations due to well-known systematic underestimation of insertions and deletions in sequencing data. Second, PyClone failed to separate the NRAS (G12V) mutation from the founding clone mutations in AML-34, despite the mutation being present in virtually all cells in eight of the nine samples but completely absent from the xenograft which instead carried the NRAS (G12S) mutation. Third, in AML-7, PyClone combined the *PTPN11* and *WT1* mutations into a single clone despite the presence of samples containing the *WT1* mutation but not the *PTPN11* mutation and vice versa. The same was done for the *NF1* and *FLT3* clones, which were anticorrelated, with PDX-7AA containing the *FLT3* clone at 14% and the *NF1* clone at 64% but PDX-7B containing the *FLT3* clone at 74% and the *NF1* clone at 29%.

Supplementary References

1 Roth, A. *et al.* PyClone: statistical inference of clonal population structure in cancer. *Nature methods* **11**, 396-398, doi:10.1038/nmeth.2883 (2014).
