# Supplementary material for: Clonal competition within complex evolutionary hierarchies shapes AML over time
Source: Nat Commun. 2020 Feb 5;11:579. doi: 10.1038/s41467-019-14106-0 (PMC7002407; doi:10.1038/s41467-019-14106-0)
Supplement: Supplementary file 3 — Description of Additional Supplementary Files [file 41467_2019_14106_MOESM3_ESM.pdf]

Supplementary Data Legends

File name: Supplementary Data 1 (AML-7)
Description: PyClone-generated clonal composition based on all detected mutations in AML-7 and corresponding xenografts.

File name: Supplementary Data 2 (AML-9)
Description: PyClone-generated clonal composition based on all detected mutations in AML-9 and corresponding xenografts.

File name: Supplementary Data 3 (AML-11)
Description: PyClone-generated clonal composition based on all detected mutations in AML-11 and corresponding xenografts.

File name: Supplementary Data 4 (AML-16)
Description: PyClone-generated clonal composition based on all detected mutations in AML-16 and corresponding xenografts.

File name: Supplementary Data 5 (AML-17)
Description: PyClone-generated clonal composition based on all detected mutations in AML-17 and corresponding xenografts.

File name: Supplementary Data 6 (AML-20)
Description: PyClone-generated clonal composition based on all detected mutations in AML-20 and corresponding xenografts.

File name: Supplementary Data 7 (AML-21)
Description: PyClone-generated clonal composition based on all detected mutations in AML-21 and corresponding xenografts.

File name: Supplementary Data 8 (AML-24)
Description: PyClone-generated clonal composition based on all detected mutations in AML-24 and corresponding xenografts.

File name: Supplementary Data 9 (AML-25)
Description: PyClone-generated clonal composition based on all detected mutations in AML-25 and corresponding xenografts.

File name: Supplementary Data 10 (AML-26)
Description: PyClone-generated clonal composition based on all detected mutations in AML-26 and corresponding xenografts.

File name: Supplementary Data 11 (AML-27)
Description: PyClone-generated clonal composition based on all detected mutations in AML-27 and corresponding xenografts.

File name: Supplementary Data 12 (AML-28)

Description: PyClone-generated clonal composition based on all detected mutations in AML-28 and corresponding xenografts.

File name: Supplementary Data 13 (AML-33)
Description: PyClone-generated clonal composition based on all detected mutations in AML-33 and corresponding xenografts.

File name: Supplementary Data 14 (AML-34)
Description: PyClone-generated clonal composition based on all detected mutations in AML-34 and corresponding xenografts.

File name: Supplementary Data 15 (AML-37)
Description: PyClone-generated clonal composition based on all detected mutations in AML-37 and corresponding xenografts.

File name: Supplementary Data 16 (AML-48)
Description: PyClone-generated clonal composition based on all detected mutations in AML-48 and corresponding xenografts.

File name: Supplementary Data 17 (AML-54)
Description: PyClone-generated clonal composition based on all detected mutations in AML-54 and corresponding xenografts.

File name: Supplementary Data 18 (AML-67)
Description: PyClone-generated clonal composition based on all detected mutations in AML-67 and corresponding xenografts.

File name: Supplementary Data 19 (AML-74)
Description: PyClone-generated clonal composition based on all detected mutations in AML-74 and corresponding xenografts.

File name: Supplementary Data 20 (AML-79)
Description: PyClone-generated clonal composition based on all detected mutations in AML-79 and corresponding xenografts.

File name: Supplementary Data 21 (AML-83)
Description: PyClone-generated clonal composition based on all detected mutations in AML-83 and corresponding xenografts.

File name: Supplementary Data 22 (AML-107)
Description: PyClone-generated clonal composition based on all detected mutations in AML-107 and corresponding xenografts.

File name: Supplementary Data 23 (AML-7)
Description: Detected somatic variants and their frequencies in AML-7 and corresponding xenografts.

File name: Supplementary Data 24 (AML-9)
Description: Detected somatic variants and their frequencies in AML-9 and corresponding xenografts.

File name: Supplementary Data 25 (AML-11)
Description: Detected somatic variants and their frequencies in AML-11 and corresponding xenografts.

File name: Supplementary Data 26 (AML-16)
Description: Detected somatic variants and their frequencies in AML-16 and corresponding xenografts.

File name: Supplementary Data 27 (AML-17)
Description: Detected somatic variants and their frequencies in AML-17 and corresponding xenografts.

File name: Supplementary Data 28 (AML-20)
Description: Detected somatic variants and their frequencies in AML-20 and corresponding xenografts.

File name: Supplementary Data 29 (AML-21)
Description: Detected somatic variants and their frequencies in AML-21 and corresponding xenografts.

File name: Supplementary Data 30 (AML-24)
Description: Detected somatic variants and their frequencies in AML-24 and corresponding xenografts.

File name: Supplementary Data 31 (AML-25)
Description: Detected somatic variants and their frequencies in AML-25 and corresponding xenografts.

File name: Supplementary Data 32 (AML-26)
Description: Detected somatic variants and their frequencies in AML-26 and corresponding xenografts.

File name: Supplementary Data 33 (AML-27)
Description: Detected somatic variants and their frequencies in AML-27 and corresponding xenografts.

File name: Supplementary Data 34 (AML-28)
Description: Detected somatic variants and their frequencies in AML-28 and corresponding xenografts.

File name: Supplementary Data 35 (AML-33)
Description: Detected somatic variants and their frequencies in AML-33 and corresponding xenografts.

File name: Supplementary Data 36 (AML-34)
Description: Detected somatic variants and their frequencies in AML-34 and corresponding xenografts.

File name: Supplementary Data 37 (AML-37)
Description: Detected somatic variants and their frequencies in AML-37 and corresponding xenografts.

File name: Supplementary Data 38 (AML-48)
Description: Detected somatic variants and their frequencies in AML-48 and corresponding xenografts.

File name: Supplementary Data 39 (AML-54)
Description: Detected somatic variants and their frequencies in AML-54 and corresponding xenografts.

File name: Supplementary Data 40 (AML-67)
Description: Detected somatic variants and their frequencies in AML-67 and corresponding xenografts.

File name: Supplementary Data 41 (AML-74)
Description: Detected somatic variants and their frequencies in AML-74 and corresponding xenografts.

File name: Supplementary Data 42 (AML-79)
Description: Detected somatic variants and their frequencies in AML-79 and corresponding xenografts.

File name: Supplementary Data 43 (AML-83)
Description: Detected somatic variants and their frequencies in AML-83 and corresponding xenografts.

File name: Supplementary Data 44 (AML-107)
Description: Detected somatic variants and their frequencies in AML-107 and corresponding xenografts.

File name: Supplementary Data 45 (AML-11)
Description: Detected copy-number aberrations in AML-11 and corresponding xenografts.

File name: Supplementary Data 46 (AML-16)
Description: Detected copy-number aberrations in AML-16 and corresponding xenografts.

File name: Supplementary Data 47 (AML-21)
Description: Detected copy-number aberrations in AML-21 and corresponding xenografts.

File name: Supplementary Data 48 (AML-25)

Description: Detected copy-number aberrations in AML-25 and corresponding xenografts.

File name: Supplementary Data 49 (AML-26)
Description: Detected copy-number aberrations in AML-26 and corresponding xenografts.

File name: Supplementary Data 50 (AML-28)
Description: Detected copy-number aberrations in AML-28 and corresponding xenografts.

File name: Supplementary Data 51 (AML-33)
Description: Detected copy-number aberrations in AML-33 and corresponding xenografts.

File name: Supplementary Data 52 (AML-34)
Description: Detected copy-number aberrations in AML-34 and corresponding xenografts.

File name: Supplementary Data 53 (AML-37)
Description: Detected copy-number aberrations in AML-37 and corresponding xenografts.

File name: Supplementary Data 54 (AML-48)
Description: Detected copy-number aberrations in AML-48 and corresponding xenografts.

File name: Supplementary Data 55 (AML-54)
Description: Detected copy-number aberrations in AML-54 and corresponding xenografts.

File name: Supplementary Data 56 (AML-74)
Description: Detected copy-number aberrations in AML-74 and corresponding xenografts.

File name: Supplementary Data 57 (AML-83)
Description: Detected copy-number aberrations in AML-83 and corresponding xenografts.

File name: Supplementary Data 58 (AML-107)
Description: Detected copy-number aberrations in AML-107 and corresponding xenografts.
